# Dysregulated mitophagy and mitochondrial organization in optic atrophy due to *OPA1* mutations

**DOI:** 10.1212/WNL.0000000000003491

**Published:** 2017-01-10

**Authors:** Chunyan Liao, Neil Ashley, Alan Diot, Karl Morten, Kanchan Phadwal, Andrew Williams, Ian Fearnley, Lyndon Rosser, Jo Lowndes, Carl Fratter, David J.P. Ferguson, Laura Vay, Gerardine Quaghebeur, Isabella Moroni, Stefania Bianchi, Costanza Lamperti, Susan M. Downes, Kamil S. Sitarz, Padraig J. Flannery, Janet Carver, Eszter Dombi, Daniel East, Matilde Laura, Mary M. Reilly, Heather Mortiboys, Remko Prevo, Michelangelo Campanella, Matthew J. Daniels, Massimo Zeviani, Patrick Yu-Wai-Man, Anna Katharina Simon, Marcela Votruba, Joanna Poulton

**Affiliations:** Author affiliations are provided at the end of the article.

## Abstract

**Objective:**

To investigate mitophagy in 5 patients with severe dominantly inherited optic atrophy (DOA), caused by depletion of OPA1 (a protein that is essential for mitochondrial fusion), compared with healthy controls.

**Methods:**

Patients with severe DOA (DOA plus) had peripheral neuropathy, cognitive regression, and epilepsy in addition to loss of vision. We quantified mitophagy in dermal fibroblasts, using 2 high throughput imaging systems, by visualizing colocalization of mitochondrial fragments with engulfing autophagosomes.

**Results:**

Fibroblasts from 3 biallelic *OPA1*(−/−) patients with severe DOA had increased mitochondrial fragmentation and mitochondrial DNA (mtDNA)–depleted cells due to decreased levels of OPA1 protein. Similarly, in siRNA-treated control fibroblasts, profound *OPA1* knockdown caused mitochondrial fragmentation, loss of mtDNA, impaired mitochondrial function, and mitochondrial mislocalization. Compared to controls, basal mitophagy (abundance of autophagosomes colocalizing with mitochondria) was increased in (1) biallelic patients, (2) monoallelic patients with DOA plus, and (3) *OPA1* siRNA–treated control cultures. Mitophagic flux was also increased. Genetic knockdown of the mitophagy protein ATG7 confirmed this by eliminating differences between patient and control fibroblasts.

**Conclusions:**

We demonstrated increased mitophagy and excessive mitochondrial fragmentation in primary human cultures associated with DOA plus due to biallelic *OPA1* mutations. We previously found that increased mitophagy (mitochondrial recycling) was associated with visual loss in another mitochondrial optic neuropathy, Leber hereditary optic neuropathy (LHON). Combined with our LHON findings, this implicates excessive mitochondrial fragmentation, dysregulated mitophagy, and impaired response to energetic stress in the pathogenesis of mitochondrial optic neuropathies, potentially linked with mitochondrial mislocalization and mtDNA depletion.

Autosomal dominant optic atrophy (DOA) is the commonest autosomal form of mitochondrial optic neuropathy, with most patients harboring pathogenic mutations in the optic atrophy 1 (*OPA1*) gene. *OPA1* mutations cause dominantly inherited progressive visual failure in the first 2 decades, secondary to optic nerve neurodegeneration. Strikingly, a subgroup of patients develops a multisystemic neurologic phenotype, known as DOA plus. Other obligate *OPA1* mutation carriers are visually asymptomatic. The mode of inheritance is autosomal dominant in the majority of cases, either haploinsufficiency or dominant-negative, with DOA plus patients frequently harboring missense mutations in the GTPase domain.

OPA1 appears to regulate mitochondrial quality control mediated through mitophagy,^1^ a specialized type of autophagy.^2^ Mitophagy is one among several types of mitochondrial quality control,^3^ and the only pathway known to turn over whole mitochondrial genomes. It is crucial for normal development^4^ and allows dysfunctional mitochondrial DNA (mtDNA) to be recycled instead of triggering cell death.^5^

We previously demonstrated increased mitophagy in fibroblasts from patients with Leber hereditary optic neuropathy (LHON).^6^ This was attenuated by idebenone, which conferred symptomatic improvement.^6^ To clarify whether increased mitophagy is an important feature of mitochondrial optic neuropathies, we investigated the role of *OPA1* in mitophagy in primary *OPA1* mutant fibroblasts from 5 patients in 3 families with severe DOA plus phenotypes. We also studied the effects of siRNA-mediated knockdown of *OPA1* in primary human control fibroblasts. Because OPA1 deficiency is widely expressed, fibroblasts have been extensively used to model the cellular mechanisms occurring in retinal ganglion and muscle cells in this multisystem disease.^7,8^

## METHODS

Mitophagy is a sequence of events in which a structure known as the autophagosome^9^ forms and engulfs spent mitochondria in a process facilitated by microtubule motors. The autophagosome is then transported towards the cellular microtubule-organizing center^10^ (MTOC) and fuses with lysosomes, ultimately resulting in the degradation of its enclosed cargo. We therefore quantified mitophagy by counting autophagosomes, that is, characteristic puncta positive for microtubule-associated protein 1 light chain 3 (LC3), and colocalizing with mitochondrial markers.^2^

### Standard protocol approvals, registrations, and patient consents.

#### Ethics: Patient and control fibroblast lines.

Patient and control samples were obtained with informed consent with the approval of the UK National Research Ethics Service (South Central-Berkshire and Newcastle and North Tyneside), or of the Ethical Committee of the Foundation Carlo Besta Institute of Neurology, according to the Declaration of Helsinki. Donors included 5 patients with DOA plus phenotypes, 5 other family members sharing mutant *OPA1* alleles, and 20 normal controls.

**Figure 1:**
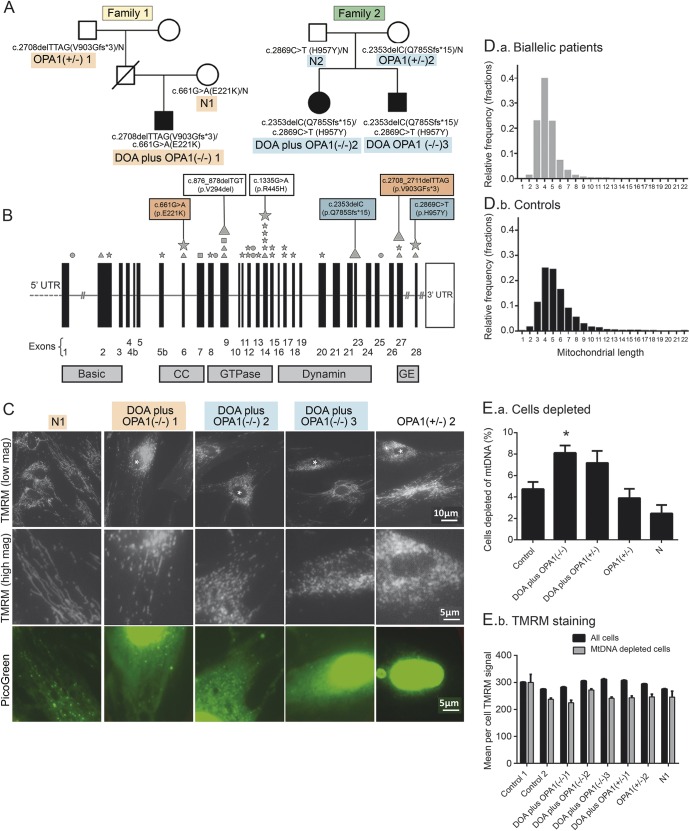
Genetic analysis of a family with a very severe dominantly inherited optic atrophy (DOA) plus phenotype (A) Pedigrees of families 1 and 2. (B) *OPA1* gene structure. Diagrammatic representation of the *OPA1* gene. The diagram indicates the location both of mutations resulting in DOA plus syndromes as described^8^ (small symbols) and of the mutations reported in this study (large symbols; highlighting corresponds to pedigree). Mutation type: stars (missense); squares (nonsense); circles (splice site); triangles (deletion). CC = coiled-coil domain; GE = GTPase effector domain; UTR = untranslated region. (C) PicoGreen/tetramethyl rhodamine methyl ester (TMRM) costaining of live fibroblasts from biallelic DOA plus *OPA1*(−/−)1–3 patients, and their symptom-free mothers (N1 and *OPA1*[+/−]2; see A). PicoGreen stains DNA and TMRM is sensitive to mitochondrial membrane potential. Nuclei of cells exhibiting mitochondrial fragmentation are marked with an asterisk. PicoGreen panel shows the same field as the high-magnification TMRM panel. TMRM staining of cells from biallelic DOA plus patients with abnormal mitochondrial fragmentation were often also depleted of mtDNA (E), but this was more marked in the siRNA-treated cell cultures in figure 2. (D) We used IN Cell 1000 to measure the mean mitochondrial length in fibroblast cultures, stained either with TMRM and PicoGreen^25,26^ or with antibody to mitochondrial protein Tom20. Cultures were grown for 3 days in 96-well plates in triplicate. To quantify the degree of mitochondrial fragmentation, we measured the average mitochondrial length in each cell and plotted a frequency distribution. This shows that while the modal length was similar in both groups, the per cell average mitochondrial length was shorter in biallelic patients (D.a) than controls (D.b) (see also figure e-3). (E) Cells depleted of mtDNA are increased (E.a) and have a lower membrane potential by TMRM staining (E.b). Error bars are 1 standard error. Asterisks indicate *p* < 0.001 compared to controls (2-tailed *t* test). Each bar represents between 400 and 1,500 cells. mtDNA = mitochondrial DNA.

Pedigrees of 3 biallelic patients harboring compound heterozygous *OPA1* mutations (strictly described as semi-dominant^11–13^) are presented in figure 1A. A summary of the clinical presentations and genotypes of all patients (illustrated in figure 1B) are presented in the table. This includes chronic progressive external ophthalmoplegia with an apparent defect in mtDNA maintenance^14,15^ that remains unexplained (DOA plus *OPA1*[+/−]1 and 2, table). Further details of the clinical presentation, a cranial MRI scan of the biallelic patients, and the likely effects on protein are presented in appendix e-1 and figure e-1, A and B, at Neurology.org. Following the convention of previous authors,^13^ we designated the 3 biallelic patients DOA plus because each had clinical and electrophysiologic evidence of both peripheral and optic neuropathy.

**Table:**
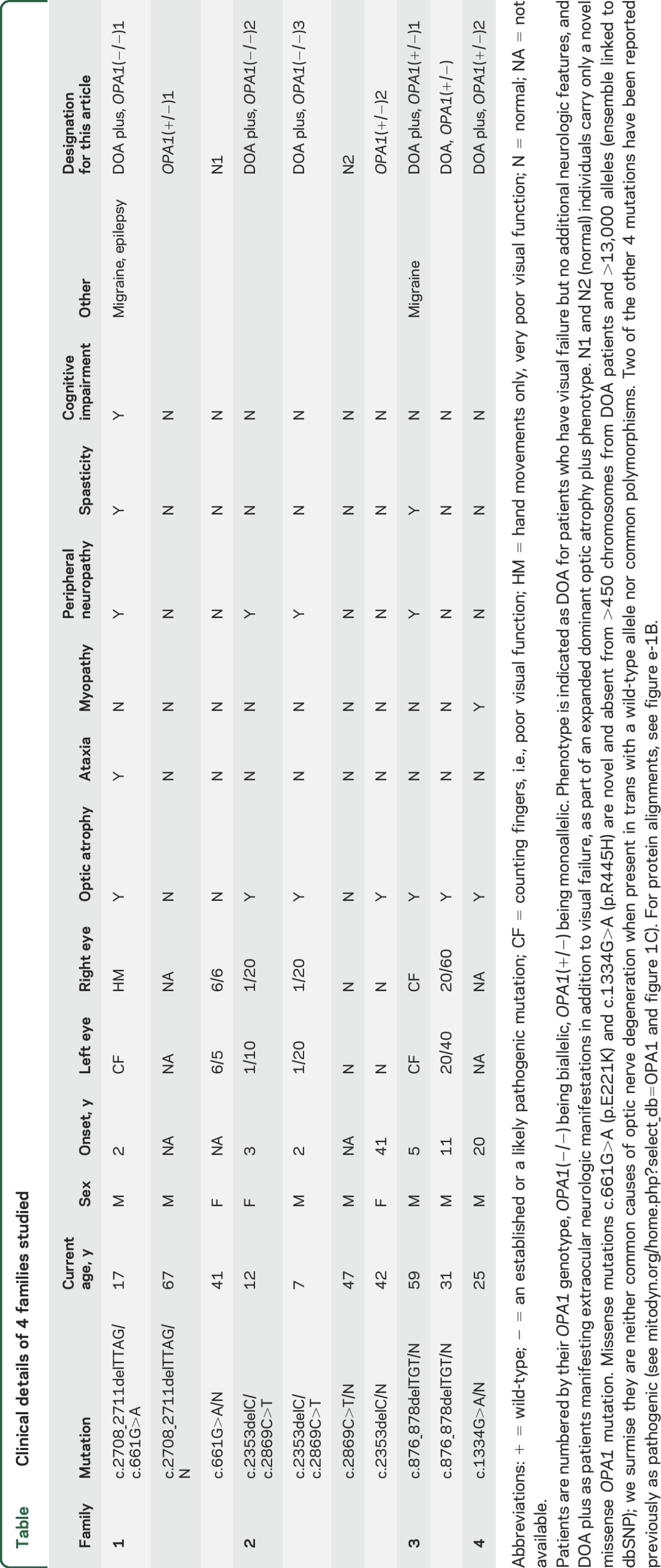
Clinical details of 4 families studied

### Immunofluorescence and live cell imaging.

Cells were processed for histochemistry, immunofluorescence, or live staining with PicoGreen and tetramethyl rhodamine methyl ester (TMRM) as previously described (appendix e-2). We used 2 high-throughput imaging systems for detecting mitophagy: the established IN Cell 1000^16^ and ImageStream, which we validated (figure e-2).

### Statistical analysis.

Statistical analysis is detailed in appendix e-2.

## RESULTS

### Biallelic *OPA1* mutant patients and families.

We studied primary fibroblasts, carrying biallelic *OPA1* mutations, from patients and transmitting relatives belonging to 2 families (see table for an explanation of nomenclature, figure 1A for pedigree, and appendix e-1 for additional clinical details). The proband of family 1, DOA plus *OPA1*(−/−)1, is a 17-year-old boy presenting with a severe *OPA1* phenotype (figure 1A). DOA plus *OPA1*(−/−)1 carries a c.2708_2711delTTAG p.V903Gfs*3 mutation, found in the paternal grandfather, in trans with a maternal c.661G>A p.E221K change (*OPA1*[+/−]1 and N1, respectively, in figure 1A). In family 2, biallelic patients DOA plus *OPA1*(−/−)2 and 3 both had a paternal c.2353delC p.Q785Sfs*15 and a maternal c.2869C>T, p.H957Y mutation (figure 1, A and B; see figure e-1B for PolyPhen analysis). No other relatives were affected. The frameshift mutation in family 1 is a well-established pathogenic mutation.^17^ None of these mutations involves the GTPase domain of *OPA1*, classically implicated in syndromic DOA,^13^ examples of which were identified in monoallelic DOA plus families 3 and 4 (table).

### Fibroblasts from DOA plus patients have a fragmented mitochondrial network with occasional mtDNA-depleted cells.

We investigated the cellular phenotype of probands, transmitting relatives, and controls. We visualized both mtDNA and mitochondria by using the DNA-specific dye PicoGreen and the mitochondrial membrane potential (MMP)–sensitive dye TMRM.^18^ The mitochondrial network had a fragmented morphology in a small minority of cells from patients DOA plus *OPA1*(−/−)1–3, but it was normal in other cells (figure 1C). Using high-throughput imaging (figure 1D), we showed that mitochondria in fibroblasts from biallelic and monoallelic DOA plus patients (DOA plus *OPA1*[−/−]1–3 and DOA plus *OPA1*[+/−]1–2) were significantly more fragmented than mitochondria from 6 controls (*p* = 0.005 and 0.01, respectively, figure e-3A). Using PicoGreen to visualize mtDNA,^19^ we found a significant increase in cells that were depleted of mtDNA in biallelic patients compared to controls with IN Cell 1000 (*p* < 0.001, figure 1E). In all cultures, these mtDNA-depleted cells had fragmented mitochondria with a lower membrane potential (figure 1E.a and 1E.b) than control cells. Intermediate mitochondrial fragmentation and mtDNA depletion were present in fibroblast cultures from DOA *OPA1*(+/−) but not from non-syndromic DOA (figure e-3A) or the asymptomatic, obligate carrier relatives of the biallelic patients.

### *OPA1* knockdown causes mtDNA depletion and alters the distribution of mitochondria in control cells.

**Figure 2:**
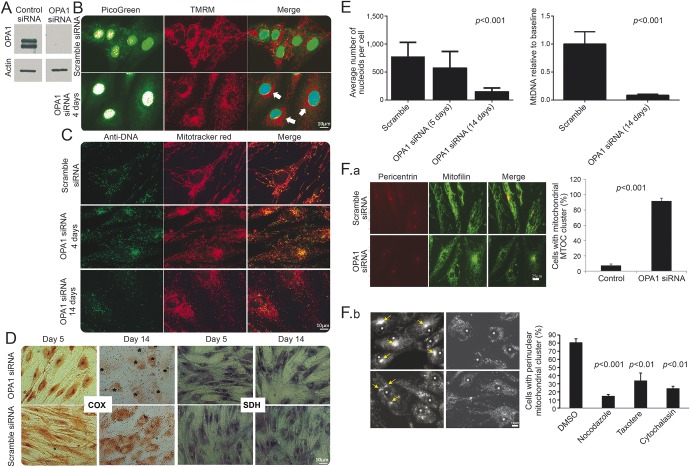
A reduction in *OPA1* leads to rapid loss of mitochondrial DNA (mtDNA) Control fibroblasts were treated with *OPA1* Pan OPA1 or scramble siRNA. (A) Western blot analysis confirms OPA1 levels were efficiently reduced following siRNA treatment (blot shows 3 days after knockdown). (B) PicoGreen/tetramethyl rhodamine methyl ester (TMRM) costaining of live fibroblasts treated with *OPA1* siRNA shows a marked loss of mtDNA nucleoids (visible as green puncta) compared to treatment with scramble. In all *OPA1* knockdown cells with mitochondrial fragmentation, a noticeable mitochondrial perinuclear clustering was observed (arrows). (C) Anti-DNA/MitoTracker colabeling of *OPA1*/scramble siRNA treated fibroblasts (note that this antibody is recognized to label mtDNA more strongly than nuclear DNA). This confirms the findings presented in B. (D) Cytochrome oxidase (COX) activity, demonstrated by routine histochemistry, is significantly reduced by day 14. In the panel showing COX at 14 days in the knockdown, the positions of nuclei are marked with asterisks. By day 5, the distribution of both COX and succinate dehydrogenase (SDH) activity reflect the mitochondrial perinuclear clustering. Figure e-6 shows that mitochondrial fragmentation occurred without loss of cytochrome *c* or alteration of cristae at 48 hours. (E) SiRNA to *OPA1* significantly reduced the number of nucleoids visualized by PicoGreen (left panel, *p* < 0.001, 2-tailed *t* test) corresponding to mtDNA content of 8% ± 2% of the control. This was confirmed by quantitative PCR 14 days after treatment (right panel, *p* < 0.001, 2-tailed *t* test). (F) Cell biological basis for relocalization of mitochondria during *OPA1* knockdown implies altered transport of fragmented mitochondria. *OPA1* knockdown relocates mitochondria to the microtubule organizing center (MTOC), mediated by microtubules and actin. That this is prevented by overexpression of p50-dynamitin is shown in figure e-3B. (F.a) Anti-pericentrin (MTOC)/mitofillin (mitochondria) staining shows that siRNA to *OPA1* causes relocation of mitochondria to the MTOC in over 90% of cells as opposed to the scrambled siRNA control (see chart). (F.b) The cell biological basis for relocalization of mitochondria during *OPA1* knockdown implies altered transport mediated by microtubules and actin. Upper left: confirms the presence of perinuclear mitochondrial clusters following *OPA1* knockdown. Upper right: Disassembly of microtubules by nocodazole treatment (5 μM, 24 hours) rescues the mitochondrial clustering (*p* < 0.001). Lower left: Taxotere (5 μM, 24 hours) causes assembly and stabilization of microtubules and hence randomly distributed mitochondrial clusters (3 clusters in one cell each marked with an arrow, *p* < 0.01). Lower right: Treatment with cytochalasin D (5 μM, 24 hours), which depolymerizes actin, also redistributes mitochondria away from the MTOC, although a weak perinuclear clustering was still present (*p* < 0.01). Each of these experiments was carried out at least 3 times.

To determine whether mitochondrial DNA depletion is a consistent effect of *OPA1* knockdown^20^ and whether it would be sufficient to affect mitochondrial function, we then knocked down *OPA1* in control fibroblasts using a pan-*OPA1*-specific siRNA,^21^ thus modeling the reduction in full-length OPA1 protein in patient cells. Compared to the reduced OPA1 protein levels seen in the patient fibroblasts, the siRNA achieved a more profound reduction (figure 2A), and knockdown cells underwent fragmentation and perinuclear clustering of the mitochondrial network (figure 2B).

Next, we visualized both mtDNA and mitochondria in the *OPA1* siRNA-treated cells,^18^ and found a marked loss of mtDNA (figure 2C). In these cells, mitochondria clustered in the perinuclear region (figure 2, B–D), and often displayed high TMRM fluorescence, suggesting increased MMP or increased organelle density. We confirmed these findings using anti-DNA immunoglobulin M/MitoTracker colabeling of mtDNA (figure 2C) and real-time PCR (figure 2E). Despite the considerable mtDNA depletion, COX activity was largely preserved at 5 days, but reduced by 14 days (figure 2D).

By using an antibody against pericentrin, we showed that the perinuclear mitochondrial clusters consistently colocalized with the MTOC (figure 2F.a). As well as being crucial for neuronal survival and function, microtubule-dependent transport mediates efficient encounters of autophagosomes with lysosomes,^22^ which cluster near the nucleus under conditions such as nutrient deprivation.^15,23^ A similar clustering of mitochondria occurs by overexpressing tau,^24^ because tau inhibits microtubule-dependent plus-end-directed transport of mitochondria. Thus, we hypothesized that clustering of mitochondria at the MTOC in knockdown cells may be due to either decreased plus-end or increased minus-end transport caused by excessive fragmentation and mitophagy. To test this idea, we exposed cells to microtubule-disrupting drugs. Nocodazole, which disassembles microtubules, rescued the perinuclear clustering so that the distribution of mitochondria resembled that in control cells (figure 2F.b). Exposure to taxotere (disrupts MTOC) and cytochalasin D (depolymerizes actin) disrupted perinuclear mitochondrial clustering, supporting our assertion that it depends on microtubules and MTOC. For a more detailed explanation, see figure e-3B. Together, these results demonstrate that *OPA1* knockdown in primary human fibroblasts causes disruption of the mitochondrial network, partial mtDNA depletion, and microtubule-dependent rearrangement of the mitochondrial distribution.

### High-throughput imaging shows that patient fibroblasts harbor increased autophagosomes colocalizing with mitochondria compared to controls.

We reasoned that the depletion of mtDNA associated with *OPA1* knockdown could be due either to slowed mtDNA synthesis or to increased mtDNA turnover and therefore investigated whether *OPA1* insufficiency/dysfunction had affected mitophagy. We measured total mitochondrial autophagy irrespective of *Parkin* and *PINK1* using 2 high-throughput imaging systems, ImageStream and IN Cell 1000,^16^ which are established methods for quantifying autophagy and mitophagy. In each of these, antibodies to LC3 and Tom20 are used to immunolabel autophagosomes and mitochondria, respectively. In figure e-2D, we show that ImageStream and IN Cell 1000 techniques are comparable.

**Figure 3:**
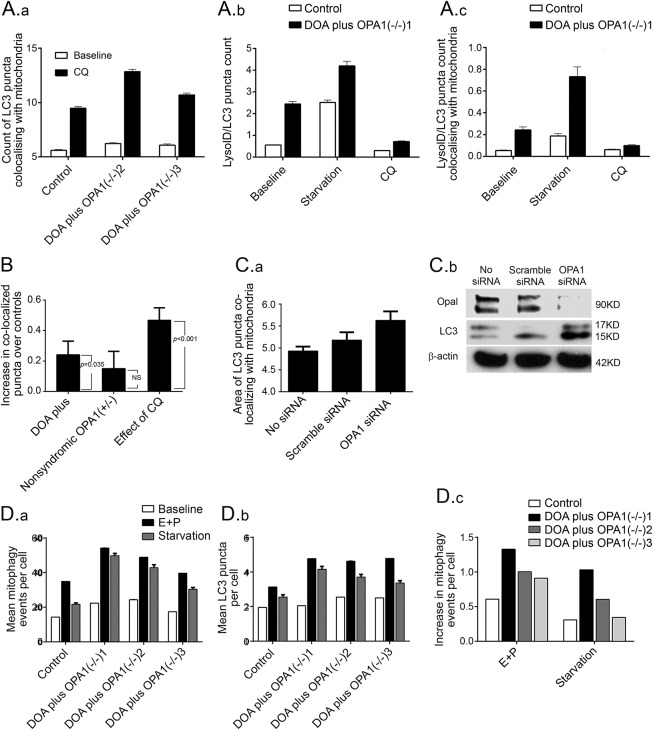
Analysis of primary cultures from biallelic dominantly inherited optic atrophy (DOA) plus *OPA1*(−/−) patients demonstrates increased mitophagy compared to an age-matched control (A) ImageStream analysis of cultured fibroblasts shows that basal mitophagy is significantly increased in DOA plus *OPA1*(−/−)2 and DOA plus *OPA1*(−/−)3 compared with control both at baseline and following treatment with CQ (all *p* < 0.01, 2-tailed *t* test). (A.a) The number of puncta per cell that were positive for light chain 3 (LC3) (representing both autophagosomes and autolysosomes) and Tom20 (representing mitochondria). These were counted at baseline in control cells, for comparison with patients DOA plus *OPA1*(−/−)2 and 3. Exposure to chloroquine (CQ; 25 μM) overnight blocks mitophagy at this stage by preventing lysosomal acidification increasing the signal, more so in patients than controls. (A.b, A.c) The counts of puncta that are positive for both LC3 and LysoID (representing only autolysosomes), counted in control cells, for comparison with patient DOA plus *OPA1*(−/−)1. (A.b) The total number of puncta per cell that were positive for the autolysosome markers. (A.c) The counts of these autolysosomes that colocalized with mitochondria (hence autolysosomes involved in mitophagy) for the same dataset. In all cases there were more counts in the patient than the control. Galactose-based starvation medium increased the number of LC3/LysoID-positive puncta above baseline. Exposure to 25 μM CQ overnight did not increase the signal, because it prevents progression of autophagosomes to autolysosomes. Error bars are standard errors (SEs) (technical replicates). Patient values all significantly greater than control *p* < 0.01 (2-tailed *t* test). All are representative of 1 out of 3 independent replicates. (B) A statistical analysis of 4 consecutive ImageStream runs on all the patients listed in the table along with 6 controls. The output shows increased mitophagy in patients with severe *OPA1* mutations (both biallelic and monoallelic, that is, DOA plus *OPA1*[−/−] and DOA plus *OPA1*[+/−]) compared with normal controls (*p* = 0.035). We show one bar per patient group, with each bar's height (y axis) representing the estimated difference between a particular patient group and controls. The whiskers on a bar represent the SE of the estimated difference (±1 SE is shown); an approximate 95% confidence interval for the patient-control difference could be calculated as the bar height ± 2 SEs. The *p* values in the figure are from the test of the null hypothesis that there is no actual difference between a patient group and controls. Useful intuition connecting the hypothesis test with the estimated difference is that a *p* value < 0.05 corresponds to a 95% confidence interval not overlapping zero. Uncomplicated symptomatic DOA *OPA1*(+/−) and asymptomatic *OPA1*(+/−) were not different from controls (n = 1, 2, and 6, respectively). Chloroquine 25 μM overnight CQ significantly increased the number of LC3 puncta colocalizing with the mitochondrial signal in all individuals in all experiments (*p* < 0.001). (C) *OPA1* knockdown by siRNA also increases mitophagy. (C.a) Bar chart of ImageStream output shows that siRNA to *OPA1* increases mitophagy. The summed area of LC3 puncta that colocalize with mitochondria (PDH signal) in fibroblasts treated with *OPA1* siRNA is greater than in scramble siRNA and the untreated controls (*p* = 0.05 and *p* < 0.01, respectively, both 2-tailed *t* tests). Mitochondrial mean intensity was also reduced by 5% (not shown). Error bars are SEs (technical replicates). (C.b) In *OPA1* knockdown fibroblasts (*OPA1* siRNA) compared to untreated (No siRNA) and siRNA scramble (Scr siRNA) controls, *OPA1* levels are reduced and LC3-II levels are substantially increased relative to actin by the Western blot analysis. (D) Validation of the increased mitophagic flux, using IN Cell 1000 quantitative fluorescence microscopy. The number of LC3-positive puncta per cell was quantitated in fibroblasts from biallelic *OPA1* patients at baseline and after 2 hours in the presence of lysosomal protease inhibitors (E64D and pepstatin A, labeled E+P) or after 2 hours starvation in minimal medium compared with age-matched controls. (D.a) Per cell count of LC3-positive puncta colocalizing with mitochondria (*p* < 0.0001 and *p* < 0.005 for baseline compared to lysosomal inhibitors or starvation, respectively). (D.b) Mean number of total LC3-positive puncta per cell. Each patient had significantly higher counts than control (*p* < 0.02) in all conditions, except baseline patient DOA plus *OPA1*(−/−)3 autophagy and baseline patient DOA plus *OPA1*(−/−)1 mitophagy. (D.c) The mitophagic flux is increased in biallelic patients relative to controls (the increase in colocalization during starvation or lysosomal inhibitors, relative to baseline). Error bars are SEs of technical replicates, all *p* values 2-tailed *t* tests. For further evidence of increased mitophagic flux, see figure e-4A.

Fibroblasts from DOA plus *OPA1*(−/−)2 and 3 (figure 3A.a) and DOA plus *OPA1*(−/−)1 (figure 3A.b and 3A.c) patients all harbored significantly more LC3-positive puncta colocalizing with mitochondrial fragments, and hence more mitophagy than those from the control using ImageStream. Colocalization of the lysosomal marker, LyosID, with LC3 puncta is used to demonstrate autolysosomes, a later stage of mitophagy than autophagosomes (figure 3A.a and 3A.b, respectively). Increased colocalization of mitochondria with LC3/LyosID-positive autolysosomes supported an increase in mitophagy in these biallelic patients (figure 3A.c). Figure 3B shows that the increase in mean level of mitophagy in the group of all DOA plus patients (combining biallelic and monoallelic) compared to controls over 4 independent experiments was significantly increased (*p* = 0.035). It was not increased in nonsyndromic monoallelic relatives. Analysis of control fibroblasts treated with *OPA1* siRNA also suggested that mitophagy was increased compared with scramble siRNA (figure 3C.a). This is consistent with the increase in LC3-II abundance on Western blot analysis (figure 3C.b).

Similarly, quantitative fluorescence microscopy using IN Cell 1000^16^ confirmed that LC3 puncta colocalizing with mitochondria were increased in cells from biallelic patients at baseline, compared to controls (figure 3D). Similar increases in basal mitophagy were seen in fibroblasts from 2 monoallelic DOA plus *OPA1*(+/−) patients who had GTPase domain mutations (DOA plus *OPA1*[+/−]1 and DOA plus *OPA1*[+/−]2), but were comparable to control levels in cells from 5 individuals who had monoallelic *OPA1* mutations (N1, N2, *OPA1*[+/−]1, *OPA1*[+/−]2, and DOA *OPA1*[+/−]).

### Mitophagic flux is increased in fibroblasts from biallelic DOA plus patients.

**Figure 4:**
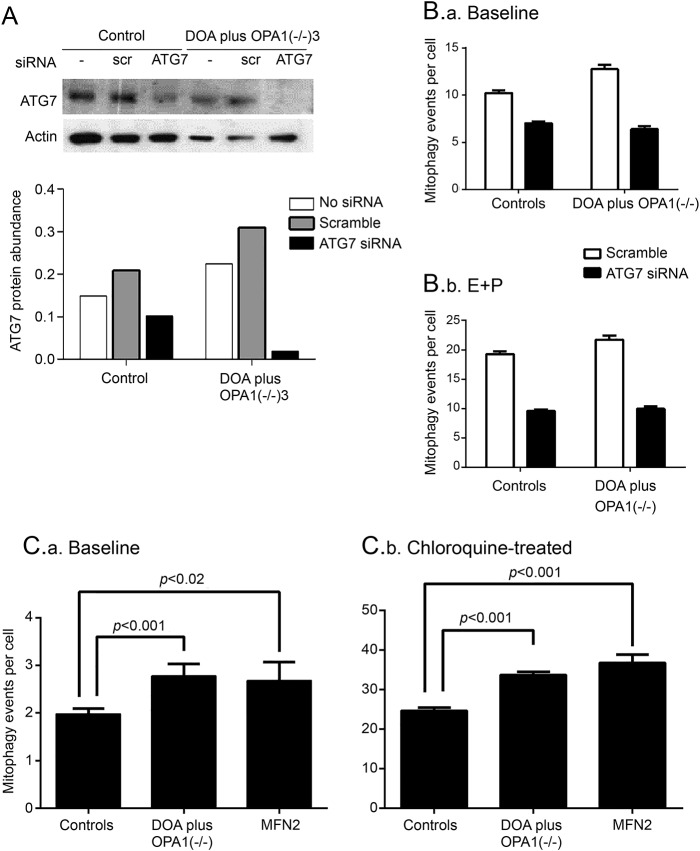
Effect of *OPA1* mutations on mitophagy is impaired by knockdown of ATG7 and recapitulated by a mitofusin 2 (*MFN2* )–dominant negative genotype (A) Western blot analysis confirms that knockdown of ATG7 by siRNA reduced protein abundance relative to actin in both patient and control cells. ATG7 = siRNA to ATG7; DOA = dominantly inherited optic atrophy; scr = scramble; siRNA = no treatment. (B) The number of colocalizing mitochondrial puncta (mitophagy events per cell, measured by IN Cell 1000) is significantly increased in fibroblasts from biallelic patients compared to controls at baseline (B.a) (*p* < 0.001) and after blocking autophagy with lysosomal inhibitors E64D and pepstatin A (E+P, *p* = 0.01). (B.b) Adding E64D and pepstatin A significantly increased the numbers of colocalizing puncta (*p* < 0.001) compared to baseline. Knockdown of ATG7 protein by siRNA significantly reduced the colocalizing puncta (*p* < 0.001) and eliminated the increase in patients over controls. Cells were treated with siRNA against *ATG7* for 48 hours, then the medium was changed and either solvent or lysosomal inhibitors, E64D, and pepstatin A were added for 3 days. Error bars are standard errors of technical replicates, all *p* values 2-tailed *t* tests. (C) The number of mitophagy events per cell (measured by IN Cell 1000) at baseline (C.a) is significantly increased in fibroblasts from biallelic DOA plus *OPA1*(−/−) patients (n = 3) and in a patient with a dominant negative *MFN2* mutation compared to control (*p* < 0.001 and *p* < 0.02, respectively). After adding chloroquine (C.b), this was significantly increased in both the DOA plus *OPA1*(−/−) patients (n = 3) and the *MFN2* fibroblasts compared to control (both *p* < 0.001). In neither case was the *MFN2* significantly different from biallelic DOA plus *OPA1*(−/−) patients. Error bars are standard errors of technical replicates, all *p* values 2-tailed *t* tests.

An increase in autophagosomes could reflect either increased autophagic activity or a reduced turnover; we therefore measured mitophagic flux. This is defined as the ratio of the magnitude of the increase in counts of puncta colocalizing with mitochondria over basal levels, relative to basal mitophagy,^2^ in a range of culture conditions and in the presence of lysosomal inhibitors. Growing fibroblasts on starvation (culture in minimal medium) or glucose-free galactose-based media (henceforth galactose medium) forces mitochondria to use oxidative phosphorylation and increases mitophagy.^16^ These culture conditions both generated a greater increase in colocalizing puncta in biallelic patients than in controls on both ImageStream (figure 3A) and IN Cell 1000 (figures 3D and 4B and not shown). Lysosomal inhibitors had a similar effect (figures 3, A, B, and D, and e-4).

Such conditions may also activate autophagy, consistent with the increase in LC3-ll seen on Western analysis of cells cultured in galactose (figure e-5), but this increase is less reproducible.

### Effect of *OPA1* mutations on mitophagy is modulated by knocking down proteins involved in mitophagy.

To confirm that the increased colocalization of LC3 puncta with mitochondria involved mitophagy, we knocked down the essential autophagy protein ATG7^4^ (figure 4A). We therefore performed RNAi on fibroblasts from DOA plus *OPA1*(−/−)1–3 patients and controls, obtaining a good reduction in ATG7 protein levels (figure 4A). Both total and colocalizing LC3 puncta were reduced by *ATG7* knockdown in all conditions (*p* < 0.001, figure 4B), eliminating the difference between biallelic patients and controls, both at baseline and after addition of the lysosomal inhibitors E64D and pepstatin A.

### Effect of idebenone.

Exposure of fibroblasts to idebenone, which modulates the increased mitophagy that we demonstrated in LHON,^6^ had no effect (figure e-4B).

### A mitofusin 2 mutation increases mitochondrial fragmentation and mitophagy.

Mitochondrial depolarization and ubiquitination are accepted triggers for mitophagy, in some situations mitophagy being amplified by ubiquitinylation of the outer membrane proteins, mitofusin 1 and 2,^25^ by Parkin, a ubiquitin ligase recruited to depolarized mitochondria in connection with PTEN-induced putative kinase 1 (PINK1).^26,27^

Neither mitochondrial depolarization nor ubiquitination were apparent in our patient fibroblasts (figure 1E and not shown), so we questioned whether mitochondrial fragmentation was sufficient in itself to trigger mitophagy. We therefore studied fibroblasts from a patient with a dominant negative mutation in another mitochondrial pro-fusion gene, mitofusin 2 (*MFN2*). These fibroblasts showed increased fragmentation of mitochondria compared to controls (*p* = 0.05), associated with increased mitophagy, both at baseline and after treatment with the lysosomal inhibitor, chloroquine (*p* < 0.02 and 0.001, respectively, figure 4C).

## DISCUSSION

We showed that profound loss of *OPA1* has several effects beyond mitochondrial fragmentation that potentially contribute to the pathogenesis of DOA and the onset of clinical disease. These include increased mitophagy, mitochondrial mislocalization, and, potentially, mitochondrial dysfunction due to mosaic mtDNA depletion.

We identified 3 patients who each carried one frameshift mutation in trans with a novel missense mutation, designated biallelic *OPA1*. The term Behr syndrome has been used for other biallelic *OPA1* families with severe phenotypes in which a missense allele, described as hypomorphic, occurs in trans with a pathogenic allele.^28^ Furthermore, both frameshift mutations caused nonsyndromic DOA with incomplete penetrance, yet caused DOA plus when combined with a missense mutation.

OPA1 is a transmembrane protein embedded within the inner mitochondrial membrane (IMM), involved in mitochondrial dynamics, specifically in IMM fusion^29^ and maintenance of cristae. It is protective against apoptosis^30^ and neurodegeneration.^31^ Mutant cells derived from patients with biallelic *OPA1* mutations not only had a lower level of OPA1 protein, but there was evidence of significant mitochondrial fragmentation compared with controls (figure 1D). A small proportion of these cells with fragmented mitochondria were profoundly depleted of mtDNA (figure 1, C and E). High-throughput quantitative imaging revealed that mitochondrial fragmentation and mtDNA depletion was also increased in monoallelic DOA plus patients with dominantly inherited *OPA1* mutations involving the GTPase domain. While *OPA1* depletion is known to cause mtDNA depletion in neurons,^32^ the association in fibroblasts is novel. In line with other investigators, fragmentation and mtDNA depletion (figure 1E) were not present in fibroblast cultures from nonsyndromic DOA patients, from the asymptomatic, obligate carrier relatives of biallelic patients, or from the controls (table).

Previous investigators found that cultured cells with even severe respiratory chain defects appear to experience rather small increases in mitophagy^33^ and that defects in respiratory chain function, if present in *OPA1* patients,^14^ are subtle.^7^ We suggest that these subtle defects may reflect the increased level of mtDNA-depleted mitochondria in cells that we documented. Two high-throughput imaging systems (ImageStream and IN Cell 1000) provide objective evidence of increased colocalization of mitochondria with autophagosomes and autolysosomes. These are more sensitive and specific for measuring mitophagy than conventional fluorescence and electron microscopy and Western blotting. Both methods showed that mitophagy is increased at baseline and following activation of autophagy in biallelic DOA plus fibroblasts, and is reduced by knockdown of the autophagy protein ATG7 (figures 4, A and B, and e-2E). The increased colocalization of mitochondria and autophagosomes represents increased mitophagic flux (figure e-4). Mitophagy was thus clearly increased in patients with monoallelic DOA plus and in severely affected biallelic *OPA1* patients, but not significantly in our monoallelic unaffected participants or in mildly affected, nonsyndromic monoallelic *OPA1* patients. The abundance of OPA1 protein reflected these differences (figure e-5). This is supported by electron microscopic findings in 2 mouse models.^17,34^

Because mitophagy does not appear to increase bulk turnover of all mitochondrial components,^35^ its importance has been called into question. It is the only type of mitochondrial quality control known to turn over whole mitochondrial genomes. While it is not clear that *OPA1* mutations directly cause mtDNA mutations or depletion, altering the dynamic cycle of mitochondrial fission and fusion is likely to dysregulate mitophagy and impair mitochondrial quality.^36^

Our data show that active mitophagy closely reflects the phenotypic severity of DOA plus due to *OPA1* depletion (figures 1E, 3B, and e-5). We suggest 3 ways in which these could be linked (figure e-7).

First, the increased mitophagy may be driven by an excess of fragmented mitochondria, potentially because of a respiratory chain defect that we did not detect. This could be beneficial or neutral. This increase is consistent with type 1 mitophagy,^37^ a subtype that is independent of *PINK1* and *Parkin*.^37^ This is because we found no evidence of increased ubiquitination (not shown) and no recruitment of the mitophagy proteins PINK1 and Parkin. It is thus plausible that increased fragmentation drives type 1 mitophagy.

Further, microtubule-dependent clustering of mitochondria, which is also apparent in *MFN2* knockdown,^38^ may also disadvantage the cell, representing a mitophagic traffic jam. For instance, clustering of fragmented mitochondria may mechanically obstruct axonal transport of functioning mitochondria or prevent mitochondrial responses to stress (stress-induced mitochondrial hyperfusion^39^).

Third, activated mitophagy may increase turnover of mitochondria and mtDNA. We showed that profound *OPA1* knockdown in control fibroblasts causes progressive loss of mtDNA and eventually mitochondrial function (figure 2E). Mitophagy may be excessive in retinal ganglion cells of *OPA1* patients, perhaps increasing demand on lysosomal pathways or causing mtDNA depletion in key locations. Indeed, *OPA1* depletion recapitulates the effects of the mitophagy-activating drug, phenanthroline. By disrupting *OPA1* processing, this metalloprotease inhibitor activates mitophagy excessively, depleting mitochondria and mtDNA and impairing the selectivity for damaged mtDNA.^16^

The interplay between these mechanisms remains to be determined (figure e-7). We showed evidence that *OPA1* depletion affects mitochondrial fragmentation, quality control, and likely microtubular transport, all important determinants of mitochondrial mass,^40^ neuronal maturation,^32^ and health.^3^ These could underline the known effects of *OPA1* depletion on neural maturation,^32^ leading to retinal ganglion cell loss, optic nerve degeneration, and hence visual failure. In particular, increased mitophagy is implicated in both LHON and syndromic parkinsonism caused by *OPA* mutations.^8^ These add biological credibility to our suggestion that dysregulated mitophagy is important in the pathogenesis of mitochondrial optic neuropathies.^6^ If so, drug modulators of mitophagy may be useful therapies for this group of disorders.

## Supplementary Material

Data Supplement

## GLOSSARY

DOA: dominantly inherited optic atrophy
IMM: inner mitochondrial membrane
LC3: light chain 3
LHON: Leber hereditary optic neuropathy
MMP: mitochondrial membrane potential
mtDNA: mitochondrial DNA
*MFN2*: mitofusin 2
MTOC: microtubule-organizing center
PINK1: PTEN-induced putative kinase 1
TMRM: tetramethyl rhodamine methyl ester
