# Supplementary material for: Dysregulated mitophagy and mitochondrial organization in optic atrophy due to OPA1 mutations
Source: Neurology. 2017 Jan 10;88(2):131–42. doi: 10.1212/WNL.0000000000003491 (PMC5224718; doi:10.1212/WNL.0000000000003491)
Supplement: Data Supplement [file supp_88_2_131__index.html]

Dysregulated mitophagy and mitochondrial organization in optic atrophy due to OPA1 mutations — Data Supplement 

# Dysregulated mitophagy and mitochondrial organization in optic atrophy due to *OPA1* mutations

## Data Supplement

**Neurology® data supplements are not copyedited before publication. Published editorials and translations have been copyedited.  
 © 2016 American Academy of Neurology.  
  
 Files in this Data Supplement:**

- Data Supplement - Microsoft Word file
