# Supplementary material for: Dysregulated mitophagy and mitochondrial organization in optic atrophy due to OPA1 mutations
Source: Neurology. 2017 Jan 10;88(2):131–42. doi: 10.1212/WNL.0000000000003491 (PMC5224718; doi:10.1212/WNL.0000000000003491)
Supplement: Data Supplement [file supp_WNL.0000000000003491_Supplemental_data.docx]

**Supplementary information**

**Appendix e-1: Supplementary clinical and molecular information (see table 1)**

**Family 1 Clinical presentation**

DOAplus OPA1(-/-)1 is a 17 year old Caucasian male from the UK, who presented with a severe phenotype due to OPA1 at the age of 10. As well as clumsiness and progressive unsteadiness, his mother reported worsening school performance and painful parasthesiae in his limbs. He admitted to loss of sensation in his hands and feet. He had developed poor vision in infancy, presenting aged 10 months with a convergent squint and corrective surgery aged 14 months. At aged 2 he was bumping into things, with progressive stepwise visual loss over 8 months. There was no relevant family history (Figure 1A).

On examination, he was a well grown boy without dysmorphic features. He had bilateral optic atrophy, but no ptosis and full eye movements. Visual acuities were finger counting in the left eye and less than this in the right. Flash electroretinograms indicated normal photoreceptor/outer retinal function, and visual evoked potentials were consistent with abnormal optic nerve conduction. Muscle bulk and power were normal, but with distal laxity in the upper limbs. In the lower limbs, he had slightly decreased ankle dorsiflexion and his reflexes were brisk with ankle clonus. He also had reduced sensation in all modalities tested, suggesting a sensory neuropathy. He was unsteady on his feet, but had no evidence of cerebellar ataxia. Cranial MRI (see below) showed very small optic nerves and chiasma. Electrophysiology showed an axonal neuropathy affecting sensory fibres more than motor. He subsequently continued to regress, developed epilepsy, and at 17 years is unable to walk independently. Neuropathic pain is now a major management problem and very recently he has developed gastrointestinal problems which are reported separately ([1](#_ENREF_1)).

Both history and clinical examination of the proband’s mother (N1, carrying c.661G>A p.E221K) and grandfather (OPA1(+/-)1 carrying c.2708_2711delTTAG OPA) were unremarkable, though the latter did not attend specialist ophthalmology appointments. The latter mutation has a known penetrance of approximately 55% ([2](#_ENREF_2)).

Mitochondrial investigations, including blood and CSF lactate, muscle biopsy COX histochemistry and respiratory chain function, were normal. Mitochondrial DNA mutations were excluded, including screening skeletal muscle for mtDNA rearrangements by LPCR and full mtDNA sequencing. MtDNA copy number in skeletal muscle was 115% of expected and in fibroblasts 94%. In addition, nuclear DNA sequencing excluded mutations in the whole of the *POLG*  and the mutation hotspot of the *PEO1* (TWINKLE) genes. The absence of mtDNA abnormalities seen in CPEO patients is probably because of his youth, and the normal respiratory chain results do not conflict with the subtle defects identified by others ([33-37](#_ENREF_33)) ([3](#_ENREF_3)).

**Family 2 Clinical presentation**

The proband, DOAplus OPA1(-/-)2, is a girl, now 12 years old, with a severe and progressive visual defect, born to non consanguineous Italian parents. Family history was negative for neurological and ophthalmologic diseases. Pregnancy and delivery were uneventful; psychomotor development was reported to be normal. Parents suspected poor vision from 3 years of age; the first ophthalmologic examination, at 6 years of age, confirmed a severe loss of visual acuity. Brain MRI was normal; visual evoked potentials revealed markedly reduced amplitudes; ERG was unremarkable. When first observed by us, at 8 years of age, the ophthalmologic evaluation revealed a marked pallor of the optic disc. Neurological examination showed bilateral *pes cavus* and reduced tendon reflexes in lower limbs, but no motor symptoms. EMG showed a reduction of Sensory Action Potentials in her lower limbs (6.0 μV in peroneal nerves) with normal Sensory Conduction Velocities.

The brother, OPA1(-/-)3, now aged 6 years and 10 months, had an identical course to his sister. He was noted to have difficulties in watching the television since the second year of life. He was examined at 4 years of age and showed the presence of similar ophthalmologic signs (reduced visual acuity, and pale optic discs at fundus exam), without signs of neurological impairment. At the last observation at 6.5 years of age he also showed bilateral *pes cavus* with mild reduction of distal tendon reflexes in his lower limbs. Plasma lactate and pyruvate were both normal in both siblings.

The neurological and ophthalmological examination of both parents was normal at first consultation (father is designated N2 and the mother OPA1(+/-)2 ). At the last examination, the mother (aged 41) showed mild signs of optic atrophy, and a pathologically thin retinal nerve fibre layer by optical coherence tomography retinal imaging. The father (aged 47) remains entirely normal. Recently a maternal aunt reported a visual defect.

**Figure e-1A: MRI scan of patient DOAplus OPA1(-/-)1**

MRI scan showed bilateral degeneration of the optic nerves (red arrows) which are very small. Proband on left, age matched “control” on right.


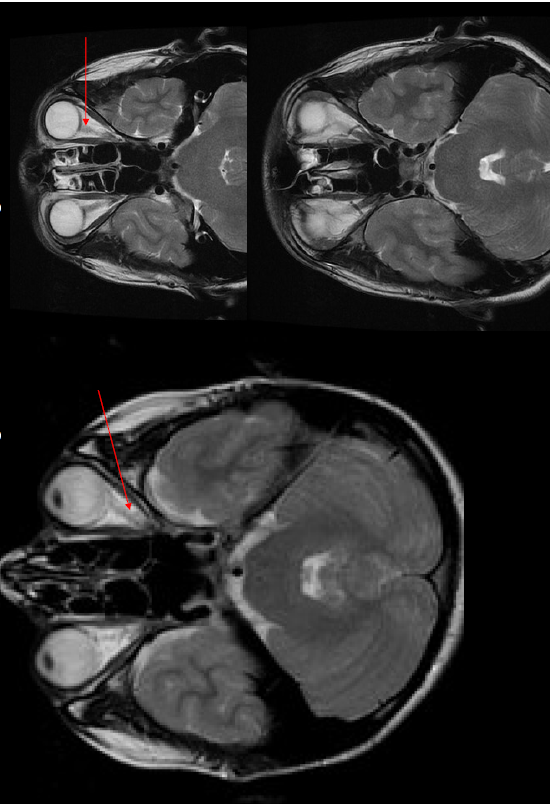


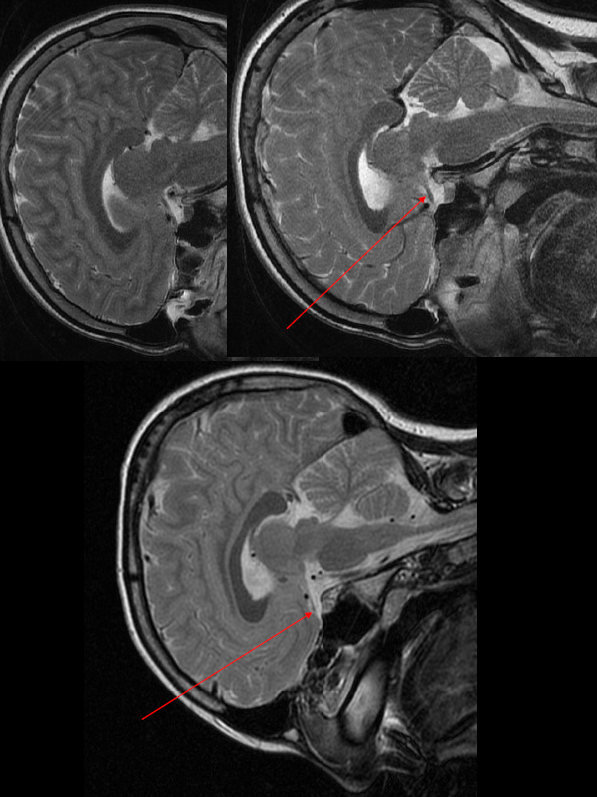
**Figure e-1B**

**Further information about OPA1 variants identified in patients whose cell cultures were analysed in detail including protein alignments.**

OPA1 sequence was used for the organisms listed, except for *D.Melanogaster, A.Gambiae* and *C.Elegans* where Opa1-like, AgaP_AGAP011286 and EAT-3 were used respectively. In *D.Rerio*, Opa1-like was used in addition to OPA1. In the bottom panel, the substituted residues are highlighted in yellow.


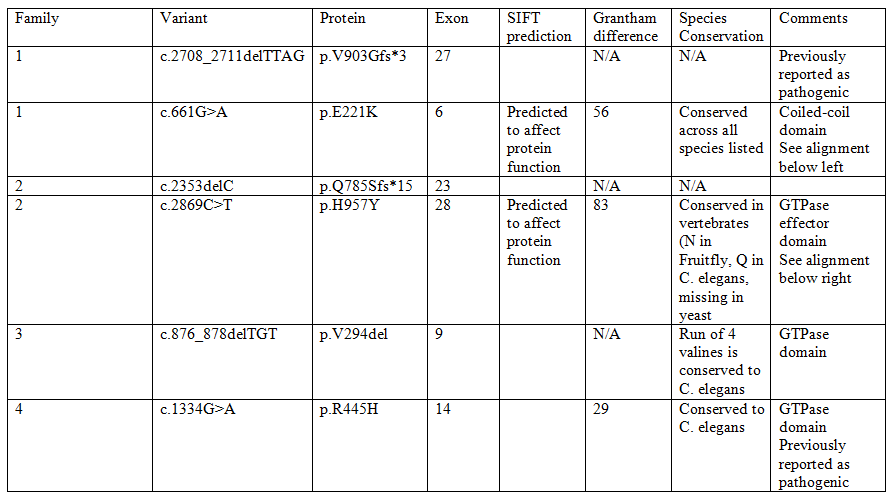


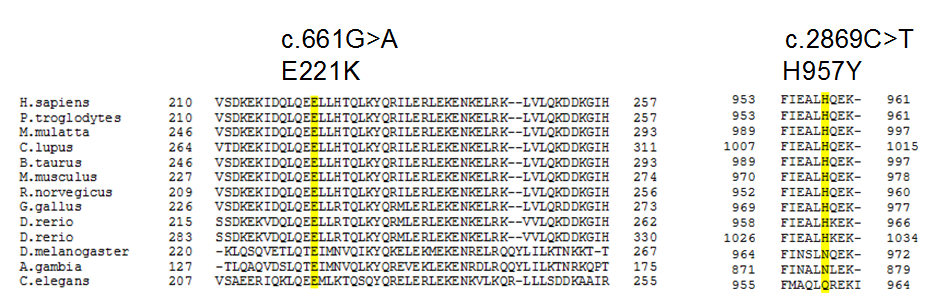


**Figure e-2. Validation of ImageStream and further validation of INCell 1000 for detecting mitophagy**

**Figure e-2A Confocal imaging of mitophagy**

Mouse embryonic fibroblasts expressing both dsred targeted to mitochondria and LC3 tagged with GFP were grown in regular medium (glucose) or in the presence of lysosomal inhibitors E64d and pepstatin A (Glucose E&P). After fixation, mitochondrial (dsred) and autophagosomes (GFP) signals were acquired on a Leica SP5 confocal microscope using a 63X lens and further digital zoom when needed. Arrowheads indicate autophagosomes engulfing mitochondria ie mitophagy (Glucose E&P zoomed in bottom panels).

**Figure e-2B Mitophagy events per cell corresponding to the confocal images in part A were quantitated using IN Cell 1000.**

This additional validation of IN Cell 1000 shows a significant increase in co-localisation of LC3 puncta with mitochondria in the presence of E64d and pepstatin A (E+P).

**Figure e-2C Raw ImageStream output** displaying 4 views on one gated cell. Channel 05 (Left) shows fibroblasts in bright field, channel 03 detects mitochondrial signal (PDH, Mitoscience) and channel 06 detects LC3 (Cell Signalling Technology), using anti-mouse Alexa-488 and anti-rabbit Alexa -546 secondary antibodies respectively (both Invitrogen). Ch03/Ch06 shows co-localisation of bright signal.

**Figure e-2D: Using IN Cell 1000 to validate ImageStream**

Cells that have been well characterised by confocal microscopy ([4](#_ENREF_4)) were used to Validate ImageStream (right chart) against IN Cell 1000 (left chart). These cells are HeLa cells expressing dsred targeted to mitochondria and GFP-tagged either autophagosome marker LC3 or lysosomal marker CD63 (courtesy of Prof A Tolkovsky ([4](#_ENREF_4))). Figures 9 and 10 of that paper ([4](#_ENREF_4)) follow mitophagy in real time by co-localisation of the mitochondrial dsred with these markers. Counts of GFP puncta (either LC3-labelled autophagosomes shown on the left of each plot, or autolysosome labelled with CD63 shown on the right of each plot) that are co-localised with mitochondria, thus reflect sequential stages of mitophagy. In corresponding ImageStream (C) and IN Cell 1000 experiments (D), the bar chart shows co-localisation of GFP puncta with mitochondrial (dsred) signal at baseline, in the presence of either 3 days glucose-free galactose media (Gal) or following 16 hours exposure to chloroquine 25 μM (Glu CQ). Chloroquine substantially increases the number of autophagosomes but not autolysosomes, galactose causes a more modest increase. The results in (C) and (D) look very similar, suggesting that both techniques are able to measure co-localisation of mitochondria with GFP puncta.

Error bars are standard errors of technical replicates.

**Figure e-2E Knock out of the essential mitophagy protein, Atg7, in mouse splenocytes reduces co-localisation of LC3 and LyosID positive puncta with mitochondria, further validating the ability of ImageStream to detect mitophagy.**

To validate that ImageStream is able to detect mitophagy we investigated mouse splenocytes, obtained from either wild type mice or from mice whose key autophagy gene, Atg7, has been excised in the haematopoietic system only (Vav-Atg7-/-) ([5](#_ENREF_5)) ([6](#_ENREF_6)).

All animal studies were carried out with approval of the Local Ethical Review Panel at the University of Oxford under license in accordance with the UK Animals (Scientific Procedures) Act 1986.

Left panel: The number of LC3 positive puncta co-localising with mitochondrial signal at baseline and after Chloroquine (25 μM chloroquine overnight) was decreased in Atg7 deficient mouse splenocytes (Atg7 KO, P<0.01).

Right panel: Puncta that were both positive for LC3 and lysosomal marker, LysoID and co-localised with mitochondria were counted to estimate the effects of Atg7 knockdown on numbers of autolysosomes. These were reduced in splenocytes from the Atg7 knockout (p<0.01). NB Chloroquine prevents lysosomal maturation by inhibiting acidification. Hence while it substantially increases the number of autophagosomes (ie of all LC3 positive puncta) co-localising with mitochondria (left) it does not much affect the counts of autolysomes co-localising with mitochondria (right).

**Figure e-2**


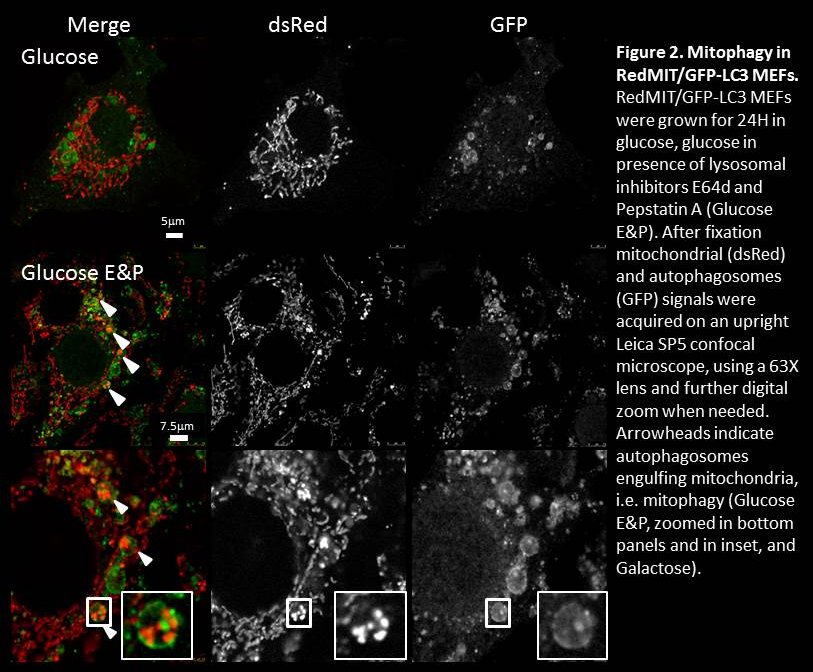


**Supplementary**

**
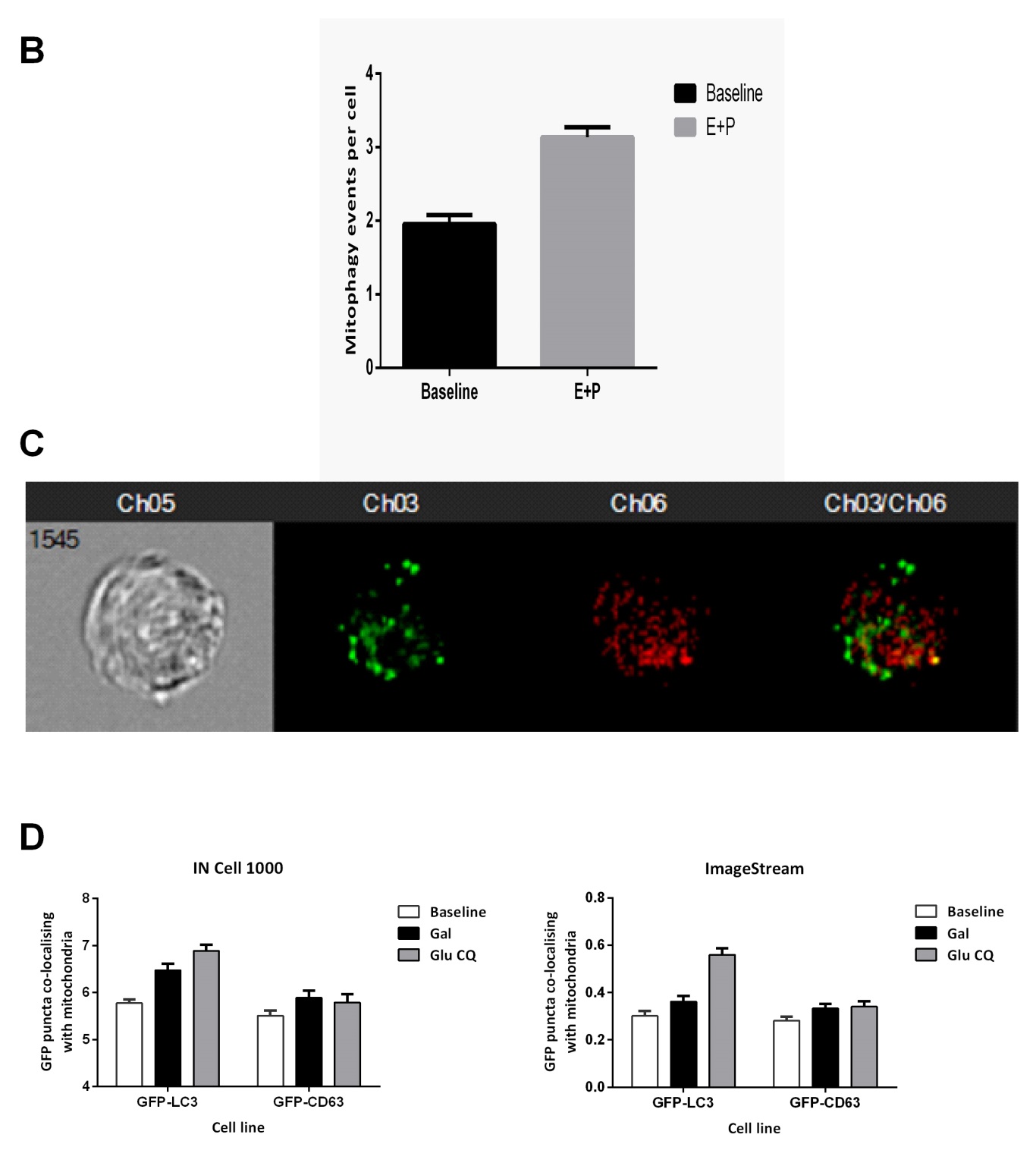
 E**


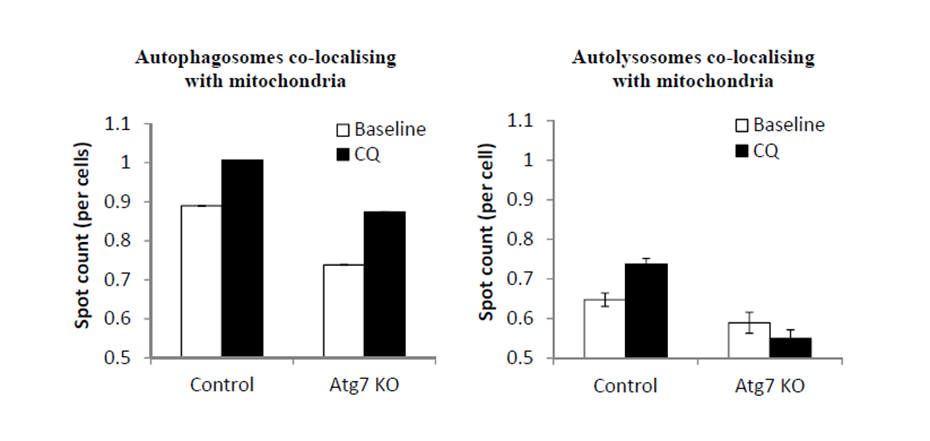


**Figure e-3.**

**Addition cellular features of OPA1 knock down.**

**Figure e-3A Confirmation of increased mitochondrial fragmentation in bi-allelic patient fibroblasts using ImageStream output, aspect ratio (the minor axis divided by the major axis, describes how round or oblong an object is, being 1.0 for a circle and lower for elongated objects) of mitochondria was determined from ImageStream output (using a mitochondrial mask based on an intensity threshold of 30%, see methods).** The difference between aspect ratio of each patient group and controls is shown as the Y axis in the chart. We show one bar per patient group, with each bar's height representing the estimated difference between a particular patient group and controls. The whiskers on a bar represent the standard error (SE) of the estimated difference (+/- 1 SE is shown); an approximate 95% confidence interval for the patient-control difference could be calculated as the bar height +/- 2 SEs. The p-values in the figure are from the test of the null hypothesis that there is no actual difference between a patient group and controls. In eight experiments involving individuals listed in Table 1 and six controls, mitochondria in fibroblasts from bi-allelic and mono-allelic DOA plus patients are significantly more fragmented than mitochondria from controls (p=0.005 and 0.01 respectively). There was no significant difference between non-syndromic patients and controls.

**Figure e-3B Microtubule dependence of the perinuclear mitochondrial clustering resulting from profound OPA1 knock down that was demonstrated in Figure 2: Dynein disruption by overexpression of p50-dynamitin-GFP rescues mitochondrial MTOC clustering due to loss of Opa1.**

We hypothesized that clustering of mitochondria at the MTOC in KD cells may be due to either an imbalance of plus- and minus-end transport caused by excessive fragmentation and mitophagy or a hitherto unknown role of Opa1 in promoting plus-end directed transport of mitochondria. In either case, loss of Opa1 would cause a preponderance of negative-end-directed transport of mitochondria along microtubules towards the MTOC, leading to perinuclear clustering. Figure 2F illustrates how we tested this idea, by exposing cells to two microtubule-disrupting drugs: nocodazole and taxotere. Nocodazole causes the disassembly of microtubules, whereas taxotere causes MTOC-independent, random assembly and stabilization of microtubules([7](#_ENREF_7)) that lack polarity and orientation([8](#_ENREF_8)). Treatment of Opa1 KD cells with nocodazole rescued the perinuclear clustering so that the distribution of mitochondria resembled that in control cells (Figure 2F(ii)). In contrast, taxotere treatment led to the formation of multiple, randomly distributed mitochondrial clusters. Finally, we also tested the role of actin filaments in Opa1 KD-mediated mitochondrial clustering by using the actin depolymerising drug cytochalasin D. As with nocodazole, treatment of KD cells with cytochalasin D caused a redistribution of mitochondria away from the MTOC, although weak perinuclear clustering was still observed in some cells. In all cases, treatment of scramble siRNA transfected cells with drugs had little effect on mitochondria (data not shown). These experiments confirm that the organization of MTOC and microtubules are pivotal to and upstream of the mitochondrial clustering caused by Opa1 KD. To determine which transport direction was involved we investigated the dynein/dynactin multi-protein complex([9](#_ENREF_9)) that mediates minus-end–directed transport of mitochondria. Overexpression of a GFP-tagged dynactin sub-unit (p50/dynamitin), disrupts the dynein/dynactin complex, leading to loss of minus-end transport([10](#_ENREF_10), [11](#_ENREF_11)), which in turn is known to disrupt autophagy([12](#_ENREF_12)),([13](#_ENREF_13)). Mitochondrial clustering was apparent with TMRM staining of OPA1 siRNA treated cells transfected with pcDNA plasmid (control, lower panel), but not in those transfected with the p50 dynamitin GFP expressing plasmid (upper panel). Nuclei are marked by asterisks. This shows that clustering is rescued by p50 dynamitin GFP expression and therefore depends on minus-end microtubule transport. Mitochondrial distribution was not affected when the same transfections were carried out with scramble siRNA treatment.

**A**

**B**


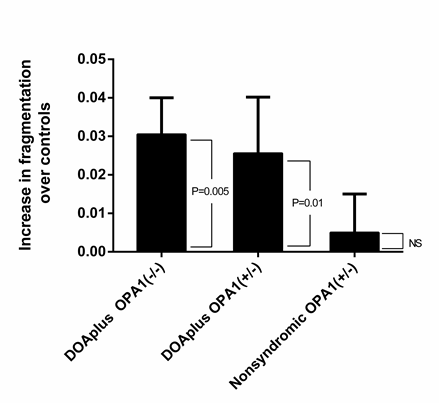


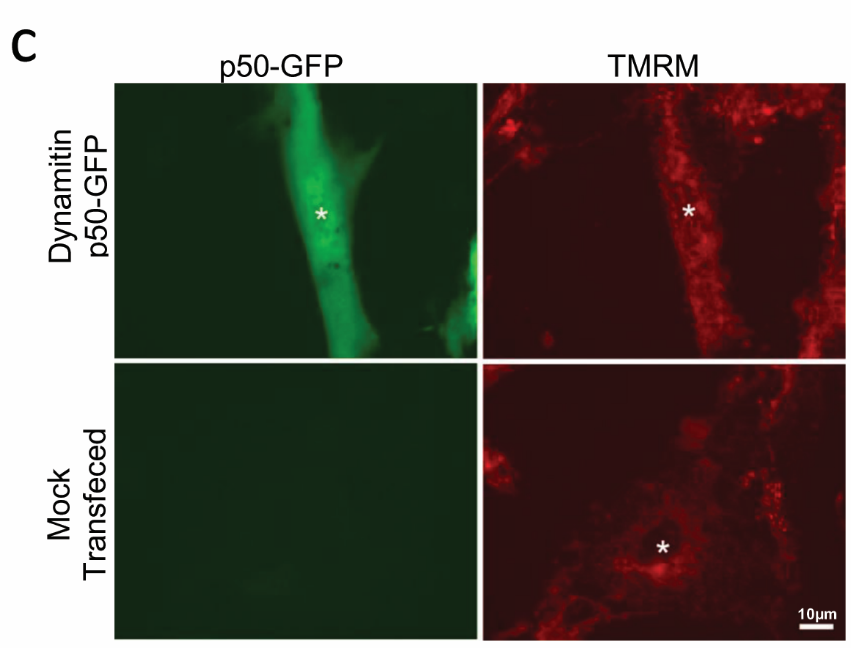


**Figure e-4**

**e-4A Mitophagic flux is increased in fibroblasts of bi-allelic patients**

Co-localisation of mitochondria with LC3 positive puncta was quantified in fibroblasts from patient DOAplus OPA1(-/-)1 and two controls using IN Cell 1000 as above. Flux (defined as the increase in co-localisation relative to baseline) was greater in the patient than the controls (p<0.001 at 24h) and increased appropriately over 24 hours.

**e-4B Idebenone does not ameliorate the increased mitophagy in fibroblasts of bi-allelic patients**

Co-localisation of mitochondria with LC3 positive puncta (expressed as mitophagy area as a percentage of mitochondrial area) in glucose-based media was not affected by 3 days exposure to idebenone 1uM for 72h (error bars are standard errors of technical replicates). This reflects idebenone treatment the animal model, in which there was no improvement ([14](#_ENREF_14)).

**Figure e-4A**

**
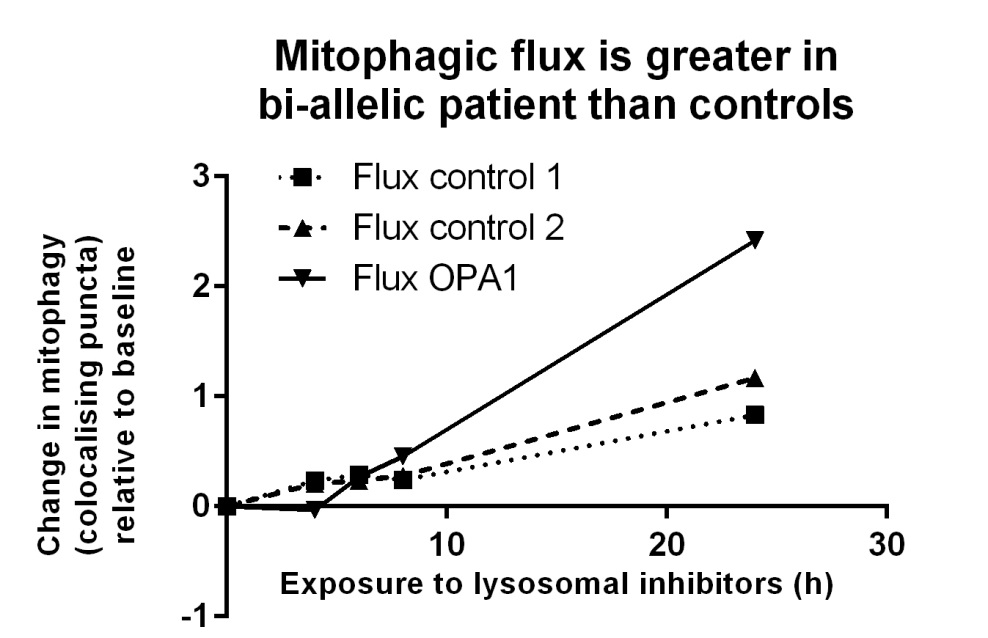
**

**Figure e-4B**

**

**

**Figure e-5 Western analysis of fibroblast protein**

A Western blot analysis of OPA1, p62 and LC3 proteins relative to actin in *OPA1* mutant fibroblasts of patients DOAplus OPA1(-/-)1-3 and mitofusin 2 (MFN2) mutant fibroblasts compared to control. Cells were grown either in regular medium (Glu) or glucose-free galactose-based medium (Gal) for 48 hours.

B The abundance of summed OPA1 short and long isoforms ([15](#_ENREF_15)) are reduced in the patients relative to the average control (Ave).

C LC3-II abundance (relative to actin) is increased in fibroblasts from patients DOAplusOPA1(-/-)2 and 3 but not DOAplusOPA1(-/-)1 compared to control

D Western blot analysis of OPA1 relative to GAPDH in *OPA1* mutant fibroblasts of patients DOAplus OPA1(+/-)1 and DOA OPA1(+/-) show a reduction in OPA1 abundance.

E Western blot analysis of OPA1 and LC3 proteins relative to actin in *OPA1* mutant fibroblasts of patient DOAplus OPA1(-/-)1 and his unaffected mother (N1) and unaffected grandfather (OPA1(+/-)1) compared to control. The lower OPA1 band correspond to the short (s-OPA1) and the upper two bands to the long (l-OPA1) isoforms ([15](#_ENREF_15)). The levels of OPA1 both short and long forms are reduced in the patient and carrier grandfather relative to the control. LC3-I and LC3-II are increased in patient 1 but not his unaffected carrier grandfather compared to control.

**Figure e-5.**


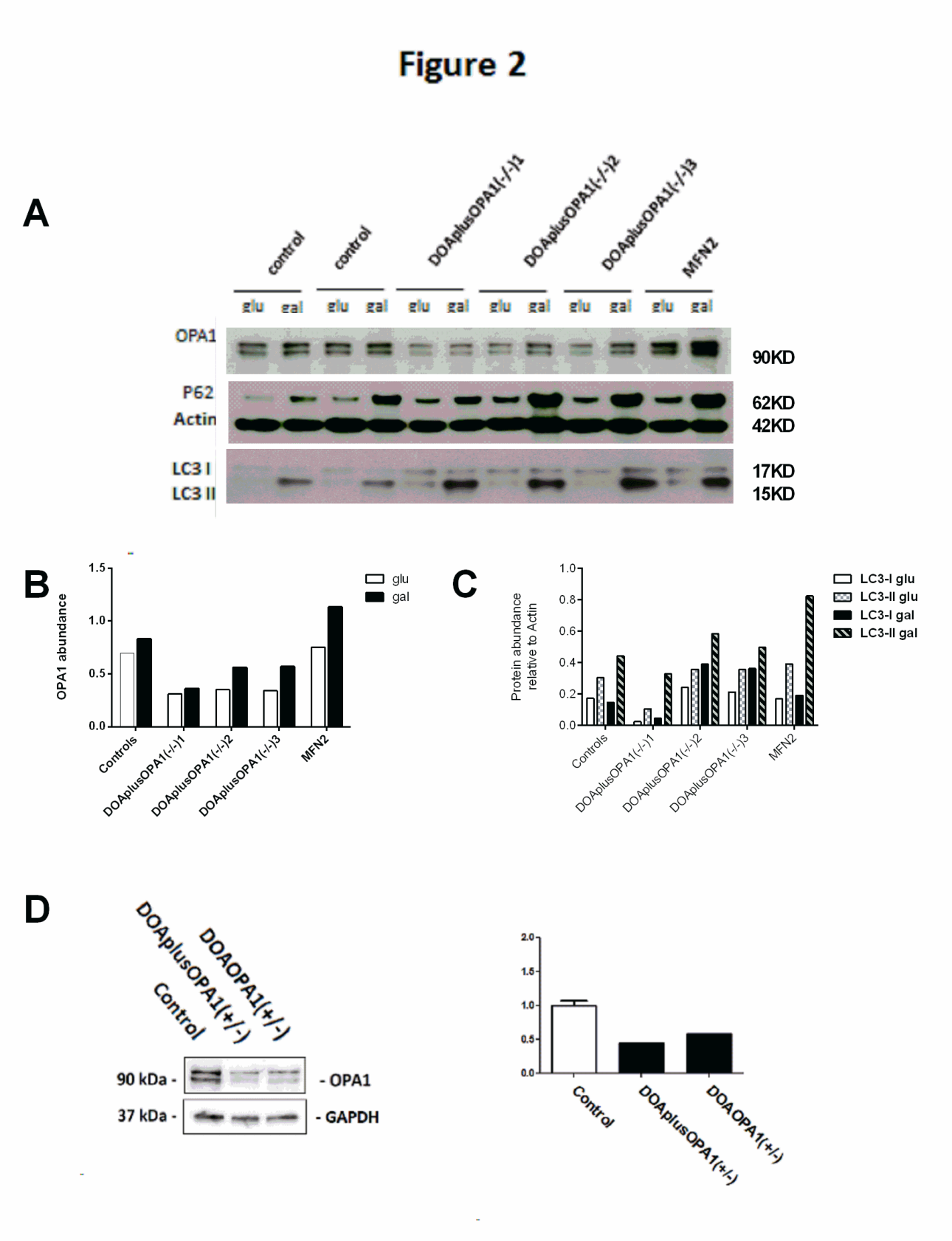


**E**


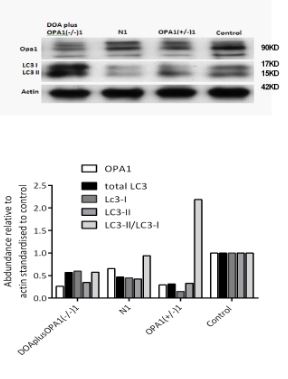


**Figure e-6. OPA1 loss in fibroblasts leads to mitochondrial fragmentation without loss of cytochrome c or alteration of cristae.**

a) Cytochrome c/Mitotracker red staining of fibroblasts treated with pan-*OPA1* or scrambled siRNA for 48 hours. There was no leakage of cytochrome c from mitochondria, [Bars 10μM]

b) Electron micrographs of mitochondria from control (left) and pan-*OPA1*/scrambled siRNA treated (right) fibroblasts showing the similar appearance of the mitochondrial cristae. [Bars 200 nm]. There was no gross increase in autophagosomes as seen by EM.


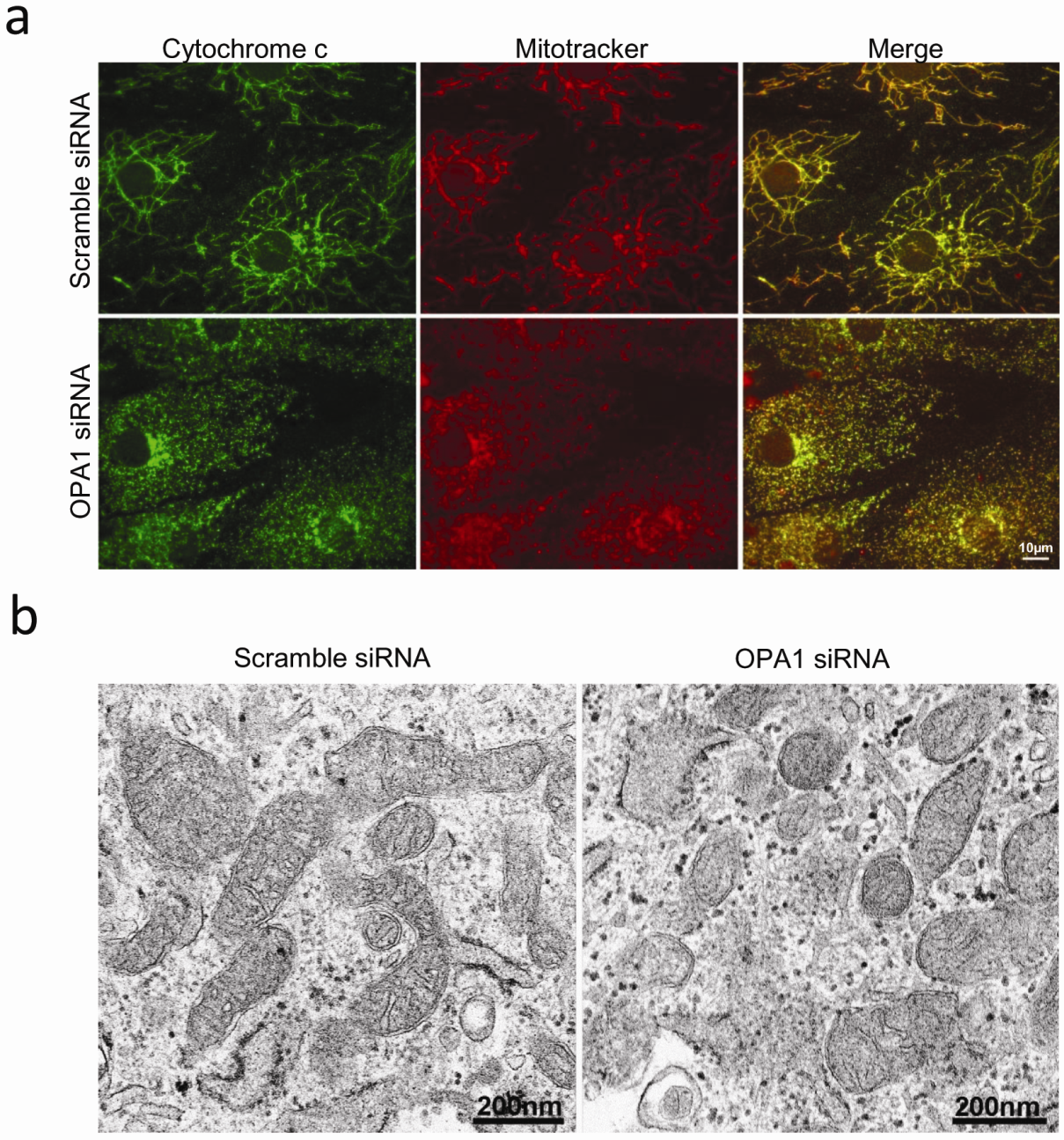


**Figure e-7**

**Illustration of suggested sequence of events**

The diagram illustrates the sequence of events that may be occurring in affected tissue with increasingly severe depletion of full length OPA1. The first four stages are apparent in fibroblasts from patients. The portion indicated by the dotted arrow illustrates our postulate that mtDNA depletion causes significant mitochondrial dysfunction in some tissues, as is apparent in siRNA of control fibroblasts. That neurodegeneration is a direct consequence of this process is unproven.

**
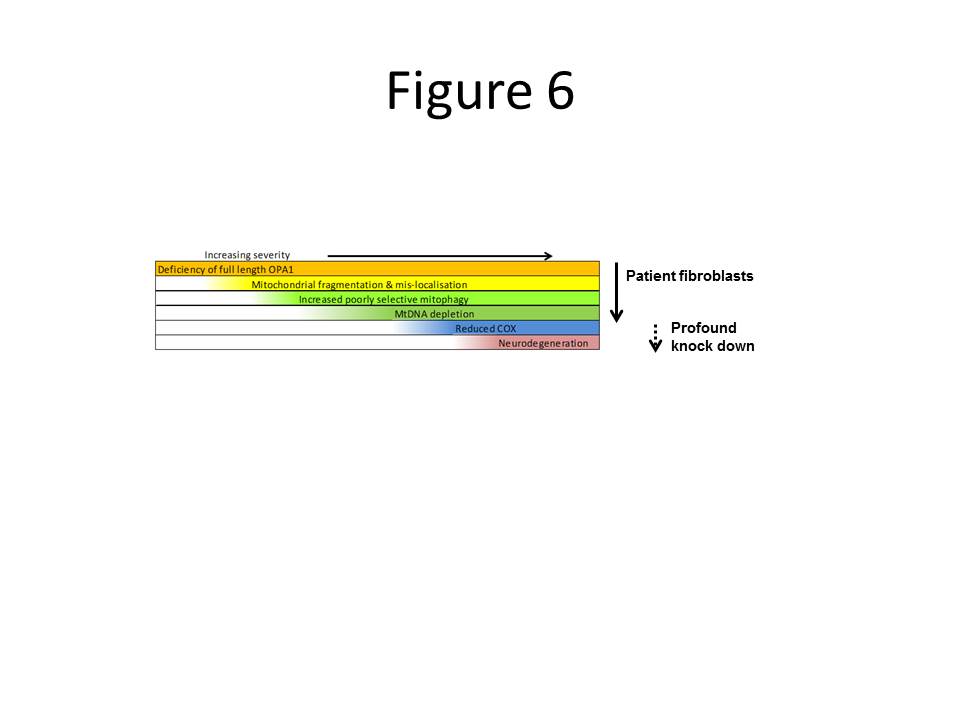
**

**Appendix e-2: Supplementary methods**

**Cell cultures** (see Table 1)

*Patient cultures*

It is generally accepted that primary patient fibroblasts express both OPA1 protein deficiency([16](#_ENREF_16)) and defects in autophagy([17](#_ENREF_17)), and are hence appropriate for pathophysiological investigations. By characterising fibroblasts from four of the healthy or oligosymptomatic transmitting relatives of the three bi-allelic patients, we were able to study each of their *OPA1* mutations in mono-allelic cultures. Three DOA patients with dominantly inherited mutations in the *OPA1* GTPase domain were studied, of whom one had uncomplicated DOA, and two had DOA plus (DOA OPA1(+/-), DOAplusOPA1(+/-)1 and DOAplus OPA1(+/-)2 respectively, see Table 1).

*Disease control culture*

One fibroblast line from a patient with severe symptomatic dominantly inherited axonal Charcot-Marie-Tooth disease CMT2A2 due to the c.745T>A (p.Ser249Thr) in the large GTPase domain near the N-terminus of mitofusin 2 was used (MFN2).

*Control cultures*

Twenty anonymised control fibroblast cultures were used for comparison (designated “control” and not including unaffected family members whose designations are in Table 1), taken (i) with parental consent from children undergoing diagnostic skin biopsy for karyotyping or biochemical screen and where cytogenetics or similar was normal (n=2), and (ii) from healthy consented adults. The age range was thus 0-81 years. One control at the median mitophagic activity, whose age was within 3 years of patient DOAplus OPA1(-/-)1 was included in the vast majority of runs.

*Cells with fluorescent organelles*

To validate our methods we also used cultured cells with fluorescent mitochondria and autophagosomes or autolysosomes. These were HeLa cells expressing dsred targeted to mitochondria and GFP tagged either LC3 or the lysosomal marker CD63 ([4](#_ENREF_4)) (courtesy of Aviva Tolkovsky). For comparison with a related defect in mitochondrial dynamics we also used cultures from a patient with MFN2 mutations.

**Immunofluorescence and live cell imaging**

Cells were processed for histochemistry([18](#_ENREF_18)), immunofluorescence or live staining with PicoGreen and TMRM as previously described([19](#_ENREF_19)). For fluorescence microscopy the antibodies used were: Anti-cytochrome c (Clone [6H2.B4](http://www.biolegend.com/index.php?page=pro_sub_cat&action=search_clone&criteria=6H2.B4), Biolegend); Anti-DNA IgM (Peter Cook); *OPA1* (Clone 18, BD Biosciences); Anti-pericentrin (ab4448, AbCam); and Anti-GM130 (clone EP892Y, AbCam). MtDNA staining in live cells was achieved by diluting stock PicoGreen solution at 3µl/ml (2 hours), then rinsed 3 times in pre-warmed PBS and mounted with phenol red free DMEM supplemented with 4.5g/l glucose and 25mM HEPES buffer, and visualised using a Leica DMI50 microscope. Mitochondrial co-labelling was achieved by incubating the cells in fresh medium containing 50nM Mitotracker red for 5 minutes before rinsing with fresh medium. Tetramethylrhodamine methyl ester (TMRM) staining was used to monitor mitochondrial membrane potential. Cells were incubated for 20 minutes in medium containing 50nM TMRM at 37^o^C and visualised without removal of the dye.

**IN Cell 1000 high throughput imaging** ([20](#_ENREF_20)).

We previously validated IN Cell 1000 for detecting mitophagy ([20](#_ENREF_20)). In brief, cells are cultured in a 96-well plate and treated for 6 hours in the indicated conditions before fixation with 4% paraformaldehyde (PFA). After DAPI and immuno-staining using a monoclonal antibody anti-TOM20 (Santa Cruz Biotechnology, mouse) and a polyclonal antibody anti-LC3 (Medical and Biological Laboratories, rabbit) combined to Alexa Fluor 488 Goat anti-rabbit and Alexa Fluor 568 Goat anti-mouse (life technologies) secondary antibodies the plate is imaged using the IN Cell1000 analyser (GE healthcare life sciences, 500 cells per well). Raw images were binarised and mitochondrial morphological characteristics were quantified, notably the degree of branching or mitochondrial form factor (FF) and the mean mitochondria length (in μm).

Figures e-2A and e-2B show a comparison of IN Cell 1000 output with confocal microscopy, using mouse embryonic fibroblasts in which dsred is targetted to mitochondria and GFP to autophagosomes by tagging LC3. Exposure to lysosomal inhibitors E64d and Pepstatin A increases the co-localisation of mitochondria with autophagosomes by both techniques, microscopy showing that the mitochondrial fragments are engulfed.

**ImageStream validation for analysis of mitophagy**

We also used ImageStream (Amnis) to quantify mitophagy in fibroblast cultures from patients and controls. This was previously validated as a method for detecting autophagy ([21](#_ENREF_21))

Primary cell cultures were harvested, washed in PBS, and fixed with 4% paraformaldehyde for 15 minutes at room temperature and permeabilized by adding 0.4% triton to the fixative for 5 minutes. They were then immunostained with antibodies to the E1 α subunit of PDH (Mitoscience) or LC3 (Cell Signalling Technology) followed by anti-mouse Alexa-488 and anti-rabbit Alexa-546 secondary antibodies respectively (Invitrogen), counting 1000-5000 cells in each condition on each of three occasions. An example of raw output from ImageStream is shown in Figure e-2C.

To identify co-localisation of autophagosomes and mitochondria as an indicator of mitophagy we used Amnis IDEAS software. Initially we quantitated bright detail similarity (BDS) of mitochondrial and LC3 signals. BDS is a feature that calculates the degree of overlapping pixel intensities taken from different channels of fluorescent imagery and is the log-transformed Pearson’s correlation coefficient that is non mean normalized. It is applied either to the open residue image or to a masked image designed to detect LC3 puncta (spots of ~1 μm with an LC3 intensity that contrasted with the rest of the cell by more that 3-6 fold basal intensity). However, we found that counting the numbers of LC3/LysoID positive puncta was more robust than using BDS, especially in runs with a relatively high background. As well as the total number of such spots per cell, we compared the number of LC3/lysoID positive puncta that co-localized with mitochondria, using a “threshold” mask detecting mitochondrial location. The threshold mask is used to exclude pixels, based on a percentage of the range of intensity values as defined by the starting mask. We routinely counted the numbers of LC3 puncta co-localising with the mitochondrial signal, defined by using thresholds at both 30% and 70% of the mitochondrial signal intensity. For these features to be accurate, it is essential to gate on cells that are bright for both fluorescent markers of interest (double positive population).

We used ImageStream for a small proportion of our experiments as we found that IN Cell 1000 was superior in its resolution, cost and reliability. Figure e-2D shows that the output of this method correlates closely with that of IN Cell 1000, which we had validated for detecting mitophagy([20](#_ENREF_20)). We further validated ImageStream for detecting mitophagy by knocking down the essential autophagy protein, Atg7 in mouse splenocytes (Figure e-2E). ImageStream and IN Cell 1000 shares a significant advantage over older methods for detecting both autophagy and mitophagy: both are high throughput techniques that are both objective and quantitative (figure e-2 compares them with confocal microscopy). However, we were unable to detect PINK or Parkin signal using either method, nor could we follow single autophagosome events in real time. To our knowledge ImageStream has not previously been used to investigate mitophagy in detail.

**Quantifying form factor and autophaghic flux with ImageStream and IN Cell 1000**

FF is defined as (Pm^2^)/(4πAm), where Pm is the length of mitochondrial outline and Am is the area of mitochondrion([22](#_ENREF_22)), and with a value comprised between 1 (fragmented network in individual dots) and 0 (infinitely connected network). Mitochondria with a FF >0.8 are fragmented and their proportion is calculated among all the mitochondria identified. For ImageStream output this was determined from output using a threshold of 30% for intensity of PDH signal.

In order to detect the autophagic flux, we used the lysosomal protease inhibitors pepstatin A and E64D, or chloroquine that blocks lysosomal acidification (either 25μM overnight or 10-100μM for 2 hours). Autophagy inducing protocols were starvation (minimal media for 2 hours), or culture in galactose-based media lacking glucose for 12-72 hours.([23](#_ENREF_23), [24](#_ENREF_24)). Autophagic flux was defined as the difference between lysosomal inhibitors added and no lysosomal inhibitors added (basal levels) relative to basal levels([24](#_ENREF_24)). So we defined mitophagic flux similarly, as the difference between mitophagy with lysosomal inhibitors added and no lysosomal inhibitors added (basal levels) relative to basal levels in mitophagy with inhibitors minus without inhibitors over basal levels (Figure 3D and figure e-2E).

Figure e-2D illustrates ImageStream output from primary cells stained for LC3 and pyruvate dehydrogenase. Starvation (either minimal or galactose medium) or the presence of lysosomal inhibitors reproducibly increased co-localisation of mitochondrial signal with LC3 puncta (Figure 3A shows patient and age-matched control in galactose medium).

Chloroquine inhibits autophagy and hence reproducibly increased the number of LC3 puncta per cell as well as bright detail similarity (BDS, a score of the co-localisation of two markers in puncta where mitochondrial and LC3 signal co-localised), co-localised spot intensity and co-localised spot count (Figure 3B, p<0.001). Furthermore it could be inhibited knock down of autophagy protein ATG7 (figure e-3A).

**Western blotting**

2x10^5^ cells were seeded in the plate and grown at 37^o^C overnight. Cells were washed twice in cold PBS and harvested in 50ul 1X Laemmli sample buffer. The cells were then lysed by pulsing the sonicator for 5 seconds on/5 seconds off, twice, heated at 95°C for 5mins, and centrifuged at 15700g for 1 minute. 10ul of the supernatant were loaded on to a 4-20% gel for SDS-PAGE. Protein was separated by electrophoresis at 125V for 2 hours (Figure 3). The LC3 antibody was from MBL International Corporation (Cat. No: PM036). The OPA1 antibody was from BD transduction laboratories (Cat. No: 612606). The P62 antibody is from BD transduction laboratories (Cat.No: 610832). The actin antibody is from cell signalling (Cat. No: 11/2011).

**Electron Microscopy**

The cell pellets were fixed in 2.5 % glutaraldehyde in 0.1M phosphate buffer, post-fixed in osmium tetroxide, dehydrated and embedded in epoxy resin. Thin sections were cut and stained with uranyl acetate and lead citrate prior to examination in a Jeol 1200EX electron microscope.

**RNAi and Plasmid Transfections**

RNAi for *OPA1* was carried out as previously ([25](#_ENREF_25)) using 50nM siRNA control and target Dharmacon 1 transfection agent (Thermo-Fisher). The *ATG7* siRNA used SMARTpool: ON-TARGETplus from Thermo scientific, Cat: L-020112-00-0005, Scramble siRNA: siGENOME Non-Targeting, Thermo scientific, Cat:# D-001210-02-05A.

**Statistical Analysis**

ImageStream output (such as the mean number of LC3 puncta co-localising with mitochondria, the number of cells and standard deviation) from eight experiments involving individuals listed in Table 1 and five controls was analysed using R version 2.15.2 (the R Foundation for Statistical Computing). The components of the regression equation were: Patient ID, run ID and patient group. Separate analyses were run by grouping patients in different ways, and for each analysis each patient group was compared to the controls. In this way we determined the relationship between genotype and cellular phenotype of the patient cultures in the presence or absence of chloroquine.

Figures 1D and 2C contain one bar per patient group, with each bar's height representing the estimated difference between a particular patient group and controls. The whiskers on a bar represent the standard error (SE) of the estimated difference (+/- 1 SE is shown); an approximate 95% confidence interval for the patient-control difference could be calculated as the bar height +/- 2 SEs.

The p-values in the figure are from the test of the null hypothesis that there is no actual difference between a patient group and controls. Useful intuition connecting the hypothesis test with the estimated difference is that a p-value < 0.05 corresponds to a 95% confidence interval not overlapping zero.

**References**

1. Chapman T, Hadley G, Fratter C, Cullen S, Bax B, Bain M, et al. Unexplained gastrointestinal symptoms: Think mitochondrial disease. Digestive and Liver Disease. 2013; 46 (1): 1-8.

2. Toomes C, Marchbank NJ, Mackey DA, Craig JE, Newbury-Ecob RA, Bennett CP, et al. Spectrum, frequency and penetrance of OPA1 mutations in dominant optic atrophy. Hum Mol Genet. 2001;10(13):1369-78.

3. Nochez Y, Arsene S, Gueguen N, Chevrollier A, Ferre M, Guillet V, et al. Acute and late-onset optic atrophy due to a novel OPA1 mutation leading to a mitochondrial coupling defect. Mol Vis. 2009;15:598-608.

4. Bampton ET, Goemans CG, Niranjan D, Mizushima N, Tolkovsky AM. The dynamics of autophagy visualized in live cells: from autophagosome formation to fusion with endo/lysosomes. Autophagy. 2005;1(1):23-36.

5. Mortensen M, Ferguson DJ, Edelmann M, Kessler B, Morten KJ, Komatsu M, et al. Loss of autophagy in erythroid cells leads to defective removal of mitochondria and severe anemia in vivo. Proc Natl Acad Sci U S A. 2010;107(2):832-7.

6. Mortensen M, Soilleux EJ, Djordjevic G, Tripp R, Lutteropp M, Sadighi-Akha E, et al. The autophagy protein Atg7 is essential for hematopoietic stem cell maintenance. J Exp Med. 2011;208(3):455-67.

7. Jordan MA, Wilson L. Use of drugs to study role of microtubule assembly dynamics in living cells. Methods Enzymol. 1998;298:252-76.

8. McNiven MA, Porter KR. Microtubule polarity confers direction to pigment transport in chromatophores. J Cell Biol. 1986;103(4):1547-55.

9. Vallee RB, Okamoto PM. The regulation of endocytosis: identifying dynamin's binding partners. Trends Cell Biol. 1995;5(2):43-7.

10. Burkhardt JK, Echeverri CJ, Nilsson T, Vallee RB. Overexpression of the dynamitin (p50) subunit of the dynactin complex disrupts dynein-dependent maintenance of membrane organelle distribution. J Cell Biol. 1997;139(2):469-84.

11. Varadi A, Johnson-Cadwell LI, Cirulli V, Yoon Y, Allan VJ, Rutter GA. Cytoplasmic dynein regulates the subcellular distribution of mitochondria by controlling the recruitment of the fission factor dynamin-related protein-1. J Cell Sci. 2004;117(Pt 19):4389-400.

12. Yamamoto M, Suzuki SO, Himeno M. The effects of dynein inhibition on the autophagic pathway in glioma cells. Neuropathology. 2010;30(1):1-6.

13. Maday S, Wallace KE, Holzbaur EL. Autophagosomes initiate distally and mature during transport toward the cell soma in primary neurons. J Cell Biol. 2012;196(4):407-17.

14. Smith TG, Seto S, Ganne P, Votruba M. A randomized, placebo-controlled trial of the benzoquinone idebenone in a mouse model of OPA1-related dominant optic atrophy reveals a limited therapeutic effect on retinal ganglion cell dendropathy and visual function. Neuroscience. 2016;319:92-106.

15. Satoh M, Hamamoto T, Seo N, Kagawa Y, Endo H. Differential sublocalization of the dynamin-related protein OPA1 isoforms in mitochondria. Biochem Biophys Res Commun. 2003;300(2):482-93.

16. Amati-Bonneau P, Milea D, Bonneau D, Chevrollier A, Ferre M, Guillet V, et al. OPA1-associated disorders: phenotypes and pathophysiology. Int J Biochem Cell Biol. 2009;41(10):1855-65.

17. Cullup T, Kho AL, Dionisi-Vici C, Brandmeier B, Smith F, Urry Z, et al. Recessive mutations in EPG5 cause Vici syndrome, a multisystem disorder with defective autophagy. Nat Genet. 2013;45(1):83-7.

18. Elson JL, Samuels DC, Johnson MA, Turnbull DM, Chinnery PF. The length of cytochrome c oxidase-negative segments in muscle fibres in patients with mtDNA myopathy. Neuromuscul Disord. 2002;12(9):858-64.

19. Ashley N, Harris D, Poulton J. Detection of Mitochondrial DNA Depletion in Living Human Cells using PicoGreen Staining. Experimental Cell Research. 2005;303:432-46.

20. Diot A, Hinks-Roberts A, Lodge T, Liao C, Dombi E, Morten K, et al. A novel quantitative assay of mitophagy: combining high content analysis fluorescence with mitochondrial DNA mutant load to identify novel pharmacological modulators of mitophagy. Pharmacological Research. 2015;100:24-35.

21. Phadwal K, Alegre-Abarrategui J, Watson AS, Pike L, Anbalagan S, Hammond EM, et al. A novel method for autophagy detection in primary cells: Impaired levels of macroautophagy in immunosenescent T cells. Autophagy. 2012;8(4).

22. Mortiboys H, Thomas KJ, Koopman WJ, Klaffke S, Abou-Sleiman P, Olpin S, et al. Mitochondrial function and morphology are impaired in parkin-mutant fibroblasts. Ann Neurol. 2008;64(5):555-65.

23. Schmid D, Pypaert M, Munz C. Antigen-loading compartments for major histocompatibility complex class II molecules continuously receive input from autophagosomes. Immunity. 2007;26(1):79-92.

24. Klionsky DJ, Baehrecke EH, Brumell JH, Chu CT, Codogno P, Cuervo AM, et al. A comprehensive glossary of autophagy-related molecules and processes (2nd edition). Autophagy. 2011;7(11):1273-94.

25. Ashley N, Poulton J. Anticancer DNA intercalators cause p53-dependent mitochondrial DNA nucleoid re-modelling. Oncogene. 2009;28(44):3880-91.
